# Protomer Formation Can Aid the Structural Identification of Caffeine Metabolites

**DOI:** 10.1021/acs.analchem.2c00257

**Published:** 2022-07-21

**Authors:** Helen Sepman, Sofja Tshepelevitsh, Henrik Hupatz, Anneli Kruve

**Affiliations:** † Department of Materials and Environmental Chemistry, Stockholm University, Svante Arrhenius väg 16, 106 91 Stockholm, Sweden; ‡ Institute of Chemistry, University of Tartu, Ravila 14a, Tartu 50411, Estonia; § Institut für Chemie und Biochemie, Freie Universität Berlin, Arnimallee 20, 14195 Berlin, Germany

## Abstract

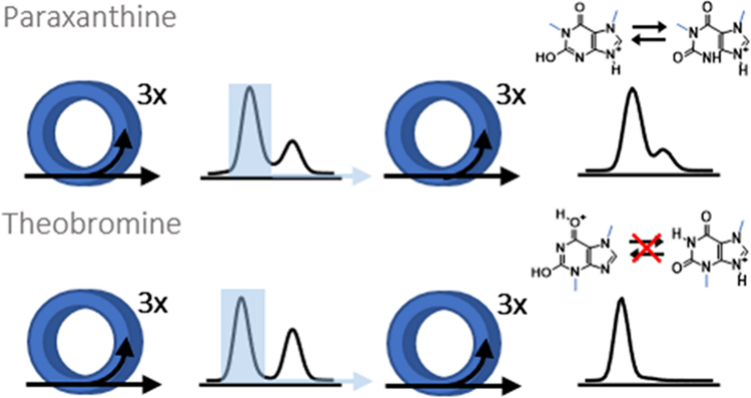

The structural annotation of isomeric metabolites remains a key challenge in untargeted electrospray ionization/high-resolution mass spectrometry (ESI/HRMS) metabolomic analysis. Many metabolites are polyfunctional compounds that may form protomers in electrospray ionization sources and therefore yield multiple peaks in ion mobility spectra. Protomer formation is strongly structure-specific. Here, we explore the possibility of using protomer formation for structural elucidation in metabolomics on the example of caffeine, its eight metabolites, and structurally related compounds. It is observed that two-thirds of the studied compounds formed high- and low-mobility species in high-resolution ion mobility. Structures in which proton hopping was hindered by a methyl group at the purine ring nitrogen (position 3) yielded structure-indicative fragments with collision-induced dissociation (CID) for high- and low-mobility ions. For compounds where such a methyl group was not present, a gas-phase equilibrium could be observed for tautomeric species with two-dimensional ion mobility. We show that the protomer formation and the gas-phase properties of the protomers can be related to the structure of caffeine metabolites and facilitate the identification of the structural isomers.

## Introduction

Accurate identification of isomeric metabolites is crucial for assessing their effect on the organism as chemically similar compounds can have a significantly different biological function.^1^ However, commonly used separation techniques do not automatically provide a possibility to identify isomeric analytes due to the similarity of chemical properties or insufficient resolving power of instruments. Despite analytical and computational developments, differentiating between chemically similar isomeric metabolites remains a challenge.^1−3^

Due to its sensitivity, selectivity, and the possibility to couple it with several separation techniques, mass spectrometry is the commonly used technique to analyze metabolites from complex samples. To produce gas-phase ions, electrospray ionization (ESI) is used in untargeted high-resolution mass spectrometry. In positive ESI mode, predominantly protonated molecules [M + H]^+^ are formed through the ion-evaporation mechanism. It has been widely debated whether the structure observed in the gas phase represents the structure of these compounds in solution.^4−12^ Coupling high-resolution mass spectrometry with ion mobility (IM) has enabled the characterization of the gas-phase structures of the formed ions and detection of so-called protomers, ions of a polyfunctional molecule where multiple sites or a chiral site can be protonated in the electrospray process.^5,10,13−16^ Therefore, multiple IM peaks with different collision cross sections may correspond to different protomers of a single compound. In several studies on polyfunctional molecules for which the most stable structures observed in the solution and gas phase differ, a strong effect of the solvent on the ratio of produced protomers has been reported.^4,10,13,17,18^ For example, it has been observed that methanol and acetonitrile can yield different protomers in ESI/MS.^18^ It has been observed that ESI source parameters such as cone voltage,^14,19,20^ solvent,^4,10,20−23^ and analyte concentration^24^ impact the protomer formation. Additionally, kinetically controlled protonation^25−27^ has been hypothesized.

Most metabolites are polyfunctional molecules prone to protomer formation, which complicates the obtained ion mobility spectra. Therefore, it has been debated that protomer formation diminishes the power of collision cross section libraries and calculated collision cross sections for the structural identification of unknown compounds.^28^ However, the possibility of obtaining additional structural information of unknown metabolomic compounds from the protomer formation has been unexplored until now.

**Figure 1:**
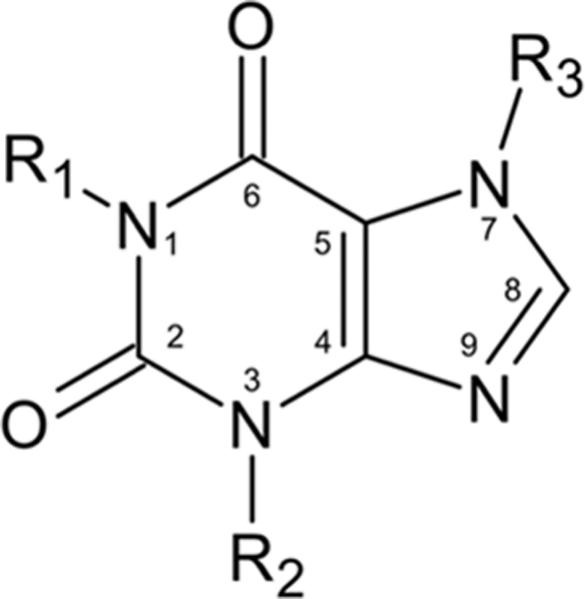
Base structure for caffeine and its metabolites (R_1_, R_2_, R_3_ = −CH_3_/–H).

We hypothesize that protomer formation can be related to specific structural features and could give characteristic information about the structure of the isomeric metabolites. To explore these possibilities, we evaluate the protomer formation of caffeine, its metabolites, and structurally related compounds with xanthine as the base structure (Figure 1) with ultrahigh resolution cyclic ion mobility spectrometry.^29^ Caffeine metabolites are polyfunctional molecules for which different protonation sites have been proposed;^30,31^ however, to the best of our knowledge, protomer formation in ESI/high-resolution mass spectrometry (HRMS) has not been reported. Additionally, we design a two-dimensional ion mobility separation approach with the possibility of further investigating the dynamic equilibrium between the protomers in the gas phase. We show that the protomer formation in combination with collision-induced dissociation/high-resolution mass spectrometry (CID/HRMS) spectra and two-dimensional ion mobility spectra can be related to the structure of the compounds and are used to discriminate between isomeric caffeine metabolites. Additionally, computational methods were used in an attempt to rationalize the experimental findings.

## Materials and Methods

### Chemicals

We acquired drift times and mass spectra for caffeine (1,3,7-trimethylxanthine, ≥99.0%, Sigma-Aldrich) and its eight metabolites theophylline (1,3-dimethylxanthine, 99.0%, Sigma-Aldrich), paraxanthine (1,7-dimethylxanthine, ∼98%, Sigma-Aldrich), theobromine (3,7-dimethylxanthine, analytical standard, Sigma-Aldrich), 1-methylxanthine (≥97.0%, Sigma-Aldrich), 3-methylxanthine (98.0%, Sigma-Aldrich), 7-methylxanthine (≥98.0%, Sigma-Aldrich), xanthine (≥99.5%, Sigma-Aldrich), and hypoxanthine (≥99.0%, Sigma-Aldrich). Additionally, drift times for guanine (98%, Alfa Aesar), adenine (high-purity grade, VWR Chemicals), thymine (97%, Alfa Aesar), and uric acid metabolites 1-methyluric acid (≥98%, Sigma-Aldrich), 1,3-dimethyluric acid (≥98%, Sigma-Aldrich), 3,7-dimethyluric acid (≥95.0%, Sigma-Aldrich), 1,7-dimethyluric acid (≥97.0%, Sigma-Aldrich), and 1,3,7,9-tetramethyluric (≥95.0%, PhytoLab) acid were acquired.

Stock solutions with concentrations in the range of 0.76–9.10 mM were prepared in a water/acetonitrile (20/80, v/v) mixture. The exceptions were xanthine, which was prepared in a methanol/ammonium hydrate (20/1, v/v) solvent mixture, as well as guanine, uric acid, and 1-methyluric acid, which were prepared in a water/ammonium hydrate 25% solution (20/1, v/v) solvent mixture. Additional 1000-fold dilutions of stock solutions in neat water, acetonitrile, and water/acetonitrile 20/80 (v/v) solvents were prepared to carry out direct infusion experiments.

To prepare solutions and carry out IMS-HRMS experiments, water (HPLC grade, Riedel-de Haën), acetonitrile (≥99.9%, Riedel-de Haën), methanol (≥99.9%, Riedel-de Haën), ammonium hydroxide (LC-MS grade, Lichropur, 25%), and formic acid (99–100%, VWR Chemicals) were used.

### Instrumentation

IMS/HRMS experiments were performed using the cyclic ion mobility (cIM) device connected to a time-of-flight (TOF) mass spectrometer with a mass resolution of 104 000 and mass accuracy of 3 ppm or less (SELECT SERIES Cyclic IMS, Waters Corporation, Milford, MA). Ion mobility spectra were acquired using three to four cycles (one cycle corresponds to the path length of 98 cm) using mass spectra in V-mode for 1 min over a range of *m*/*z* 50.0000 to 1200.0000 Da under the following experiment parameters: source temperature was 100 °C, desolvation temperature was 400 °C, the capillary voltage was optimized in the range of 1.5–2.6 kV, desolvation gas flow rate was 600 L/h, and nebulizer gas pressure was 6.0 bar. A TW velocity of 375 m/s and wave height of 10 V were used in ion mobility experiments.

IMS/CID/HRMS experiments were performed after ion mobility separation in transfer cells with collision energies of 20 and 30 V. A resolution of 13–16 was commonly observed for three passes calculated as d*t*/Δd*t*, where d*t* is the drift time of the corresponding peak and Δd*t* is the drift time difference between two separated ion mobility peaks. The IMS/CID/IMS sequence was used to perform two-dimensional ion mobility experiments (IMS^2^). During the first separation in IM, one peak was isolated from the mobilogram and injected into the prestore, while everything else was ejected. Reinjection of the isolated peak with default settings of a prearray gradient of 85 V, prearray bias of 70 V, and array offset of 45 V to IM allows us to obtain the ion mobility spectrum of the species after re-equilibration for ∼20 ms.

### Computational Data

Gibbs free energies were calculated for neutral and protonated xanthine derivatives in the gas phase and solution phases (water, acetonitrile, and water/acetonitrile (20/80, v/v) mixture).

The starting geometries of all possible protomers (including all tautomeric forms) were generated and optimized at the BP86/TZVP level of theory with the Resolution of Identity (RI) approximation. Vibrational analysis was carried out; the absence of imaginary frequencies in vibrational spectra was taken as confirmation that the geometry corresponds to energy minimum. The geometry optimization was then carried out at the same level of theory with the Conductor-Like Screening Model (COSMO), followed by single-point calculation at the BP86/TZVPD level with the Fine cavity parameter. The obtained results (total energies and surface charge density distributions) were used for the computation of Gibbs free energies of the species in solution with the Conductor-Like Screening Model for Real Solvents^32−34^ (COSMO-RS) method (at 25 °C in an infinitely dilute solution). The computations were carried out with Turbomole (V6.5^35^) and COSMOtherm^36^ software packages; see the SI for a brief description of the method.

Theoretical collisional cross sections (CCSs) with nitrogen as a buffer gas were calculated using the Ion Mobility Software (IMoS).^37,38^ We used DFT-calculated structures with NBO partial charges as input structures and applied the trajectory method for nitrogen that includes the ion quadrupole potential.

## Results

Protomer formation, its impact on fragmentation spectra, and dynamic equilibrium of the formed protomers in the gas phase were investigated for caffeine and eight of its metabolites theophylline (1,3-dimethylxanthine), paraxanthine (1,7-dimethylxanthine), theobromine (3,7-dimethylxanthine), 1-methylxanthine, 3-methylxanthine, 7-methylxanthine, xanthine, and hypoxanthine.

### Separation of Structural Isomers

The arrival time distributions (ATDs) were acquired with corresponding quadrupole-selected *m*/*z* of molecular ions for all of the studied compounds (see Table 1 for summary). With three cycles in a cyclic IM separator corresponding to a path length of 3 cm × 98 cm, it was possible to achieve close to the baseline separation of two resolvable structures for five out of eight caffeine metabolites: paraxanthine, theobromine, 1-methylxanthine, 3-methylxanthine, and 7-methylxanthine. For the two isomeric compounds, paraxanthine and theobromine, high- and low-mobility species with the same exact mass corresponding to protonated molecules were resolved; however, the third isomeric metabolite theophylline yielded one peak in the ATD, suggesting the formation of gas-phase ions with one stable protomer structure. Moreover, one peak was observed for caffeine, with a doubly methylated pyrimidine structural part similar to theophylline. For all isomeric derivatives of monomethylated xanthine, two resolved peaks were observed in ATDs, indicating the formation of two stable structural isomers.

**Table 1:**
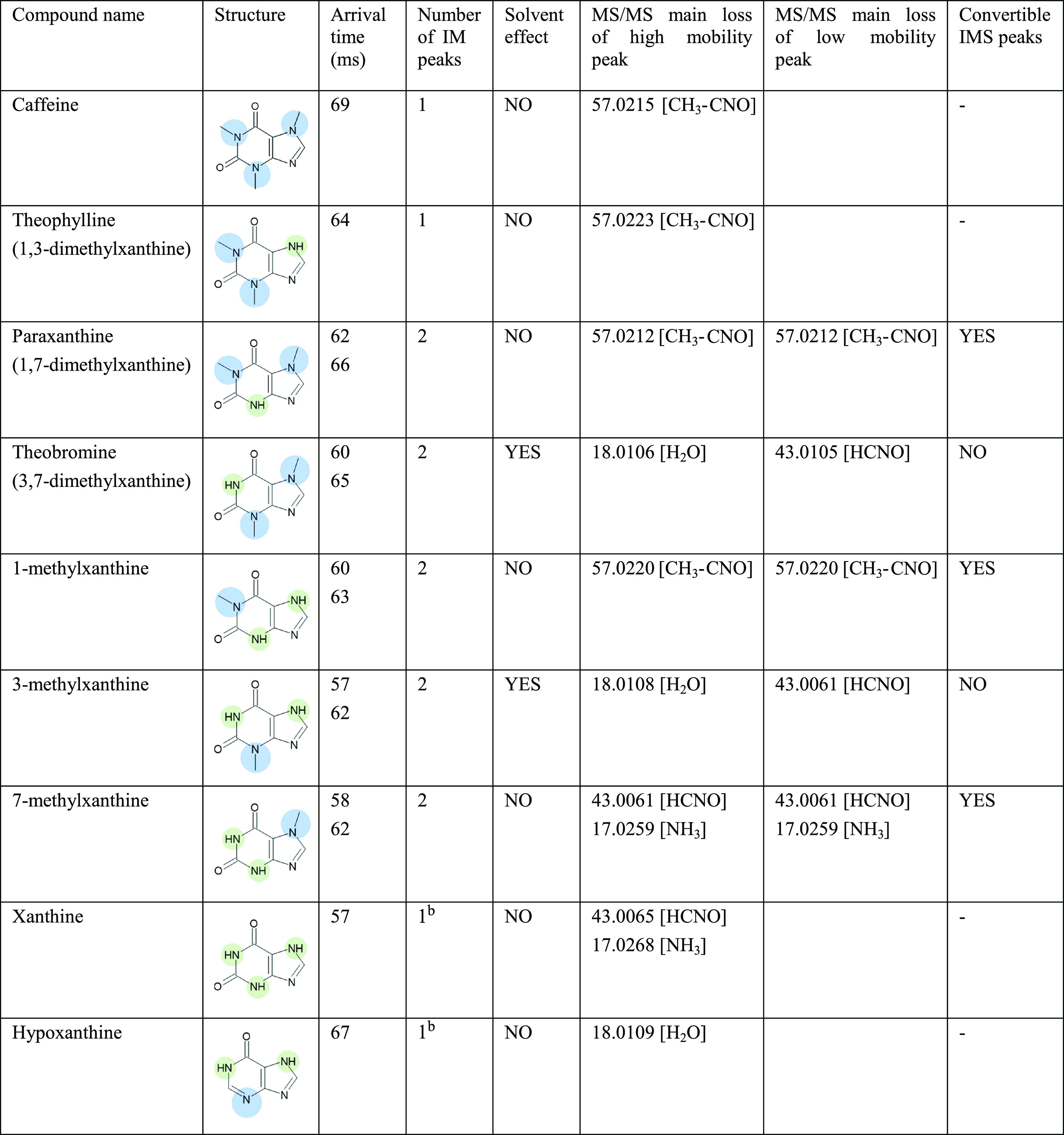
Summary Table of the Number of Peaks Observed in ATDs, Solvent Effect, and MS/MS Main Losses for Caffeine Metabolite Peaks Resolved with Cyclic Ion Mobility^a^^,^^b^

a Compounds for which solvent-affected protomers’ peaks ratio also had different fragmentation spectra for high- and low-mobility species. The structural differences, presence or absence of a methyl group, are highlighted with blue or green, accordingly.

b Number of peaks could not be identified with full confidence due to the low ionization efficiency of either the species or the substance. However, a possible second peak with low intensity could be observed.

Additionally, to assess the extent of protomer formation on species with similar structural moieties of guanine, adenine, thymine, and uric acid metabolites; 1-methyluric acid, 1,3-dimethyluric acid, 3,7-dimethyluric acid, 1,7-dimethyluric acid, and 1,3,7,9-tetramethyluric acid were investigated. For guanine and all uric acid metabolites, two or more peaks in the ATD were observed. The additional carbonyl group for uric acid derivatives provides an additional protonation site and may lead to the formation of protomer ensembles. This complicates the IM spectra while hinting toward a more complicated structure. Full details are given in Table S1.1 in the SI.

In summary, a single peak in the ATD was observed for two compounds, while for 11 compounds, two or more (partially) resolved peaks were observed. For the remaining four molecules, xanthine, hypoxanthine, adenine, and thymine, the ionization efficiency of either the substance or the second possible protomer was low; therefore, the number of peaks could not be unambiguously identified. Mobilograms acquired for all 17 substances can be found in the SI. Additionally, experimental information and results of ionization efficiency measurements can be found in the SI.

**Figure 2:**
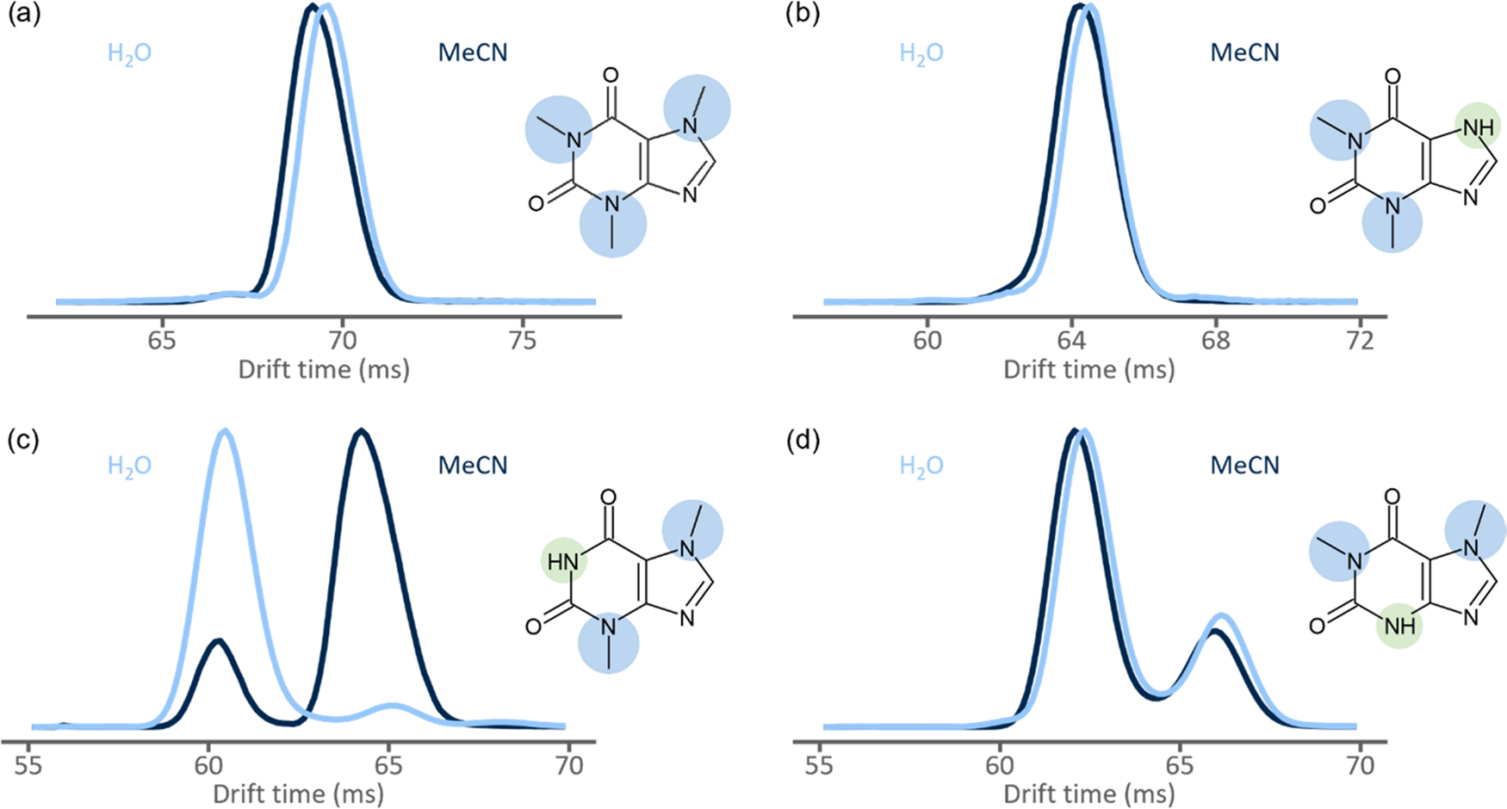
Acquired arrival time distributions (ATDs) in water (light blue) and acetonitrile (dark blue) for (a) caffeine, (b) theophylline, (c) theobromine, and (d) paraxanthine. The structural differences, presence or absence of a methyl group, are highlighted with blue or green, respectively.

Previously, protomer formation has been shown to depend on the solvent used in electrospray. Here, the ATDs were acquired both in neat water and acetonitrile and a strong solvent effect on protomer formation of theobromine and 3-methylxanthine was observed. In water solution, theobromine showed the domination of high-mobility species (peak ratio 10:1), while in acetonitrile, a higher intensity of low-mobility species was observed (peak ratio 3:10) (see Figure 2). Similarly, 3-methylxanthine followed the pattern, producing peaks with a ratio of 10:5 in water and 3:10 in acetonitrile. Generally, solvent effects are observed via so-called kinetic trapping. In this case, protonation sites in the solvent and gas phases differ, yet solvent complexes formed in the solution phase inhibit protonation to thermodynamically more favored site in the gas phase by blocking it or forbidding deprotonation.^10,14,25^ Notably, no significant change in peak ratios in ATDs was observed for other xanthine metabolites with two resolvable species. For all compounds that showed no solvent dependence in the ATD, hydrogen was present instead of the methyl group in position 3. Likewise, solvent did not affect molecules that showed one peak in ATDs.

### Effect of Protomer Formation on CID/HRMS Spectra

In the literature, caffeine metabolites are known to undergo loss of the methylisocyanate group during fragmentation;^39^ however, for some metabolites, loss of water group has additionally been suggested.^40^ Therefore, we hypothesized that the protonation site could influence the fragmentation patterns. Collision-induced dissociation (CID) for ion mobility separated high- and low-mobility species was performed to investigate this hypothesis. For all fragmentation spectra of caffeine and its metabolites, see Figures S2.1–2.13 in the SI.

**Figure 3:**
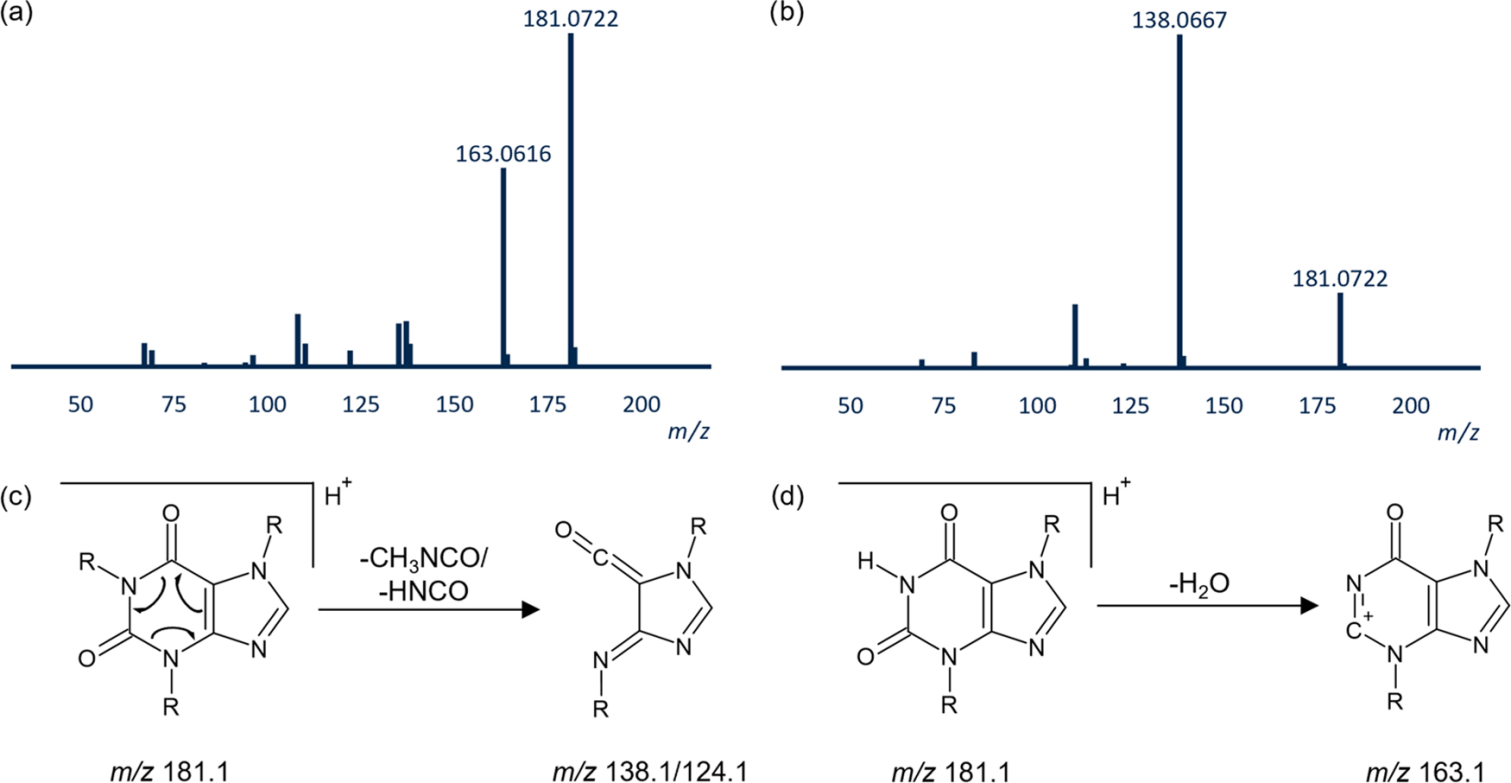
MS/MS spectra of high- and low-mobility species of theobromine (a and b, respectively) in centroid mode. The formation of main observed fragments for caffeine metabolites is shown based on the work of Bianco et al.^39^ for (c) loss of (methyl-)isocyanate and (d) loss of water.

Substances with one peak in ATDs (caffeine, theophylline) followed the proposed fragmentation pathway, loss of methylisocyanate, in all solvents. For hypoxanthine, loss of isocyanate, as well as water loss, was observed, and for xanthine, in addition to the isocyanate group, loss of NH_3_ also occurred. Fragmentation spectra of both high- and low-mobility species of paraxanthine (*m*/*z* 181.0731 C_7_H_9_N_4_O_2_) did not show any significant differences, and both species followed the proposed fragmentation pathway, forming *m*/*z* 124.0510 ([M + H – C_2_H_3_NO]^+^) as the main fragment (Figure 3). Similarly, both resolvable species for 1-methylxanthine and 7-methylxanthine resulted in similar fragmentation spectra following the expected fragmentation pattern, and the dominating fragment was produced by the loss of the (methyl-)isocyanate group. For 7-methylxanthine, additional loss of NH_3_ was observed.

For theobromine (*m*/*z* 181.0722 Da, C_7_H_9_N_4_O_2_), distinct differences in the CID/HRMS spectra of the high- and low-mobility protomers were observed. For low-mobility species, loss of the isocyanate group resulted in the domination of a fragment with an *m*/*z* of 138.0667 ([M + H – CHNO]^+^). The dominating fragment of high-mobility species was *m*/*z* 163.0616 (C_7_H_7_N_4_O), corresponding to water loss ([M + H – H_2_O]^+^) (see Figure 3). The same fragmentation pattern was observed for 3-methylxanthine. The differences in the CID/HRMS spectra for high- and low-mobility species evidence differences in the fragmentation pathways of the protomers (see Table 1 for the summary).

From arrival time distributions alone, it is not possible to deduct the exact structure, including the location of the proton, of the ion; therefore, computational methods were used to determine the thermodynamic stability of tautomeric xanthine derivatives in gas and solution phases of water, acetonitrile, and water/acetonitrile mixture (20/80, v/v) (see the SI for all results). Computed Gibbs free energies for all proposed protonated structures of caffeine metabolites are shown in the SI. For all protonated caffeine metabolites, the calculations indicate that in the gas phase, the lowest-energy tautomer is protonated at the imidazole nitrogen in position 9. Such protomers are energetically more favored by Gibbs free energy difference Δ*G* of more than 2 kcal/mol, which would make up at least 95% of the tautomeric mixture for all compounds except for 3-methylxanthine and theophylline, where the species protonated on imidazole nitrogen make up ca 68 and 94% of all of the species, respectively. This would imply that protonation should almost exclusively occur on the nitrogen atom regardless of the environment, and MS/CID/HRMS spectra would be dominated by the isocyanate loss.

Interestingly, the experimental MS/CID/HRMS spectra hint toward different protonation sites for some molecules. For example, theobromine, 3-methylxanthine, and hypoxanthine fragments [M + H – H_2_O]^+^ are observed when using a protic solvent, which suggests protonation on an oxygen atom. The differences between the lowest-energy N- and O-protonated structures in the gas phase for theobromine and 3-methylxanthine are 3.0 and 0.46 kcal/mol, respectively. For both compounds, protonation of the oxygen atom in position 6 is suggested, while amide in position 2 exhibits an iminol form. However, for hypoxanthine, O-protonated tautomers are more energetically disfavored and the energy differences with the lowest-energy N-protonated tautomer are 3 to 8 kcal/mol depending on the medium.

Based on calculations, structures protonated at the nitrogen atom in position 9 constitute over 95% of the protomers present in all media; however, most caffeine metabolites can exist in different tautomeric forms. It is important to note that we denote protomers as structures with proton/charge bound to different sites of the molecule and tautomers as rearrangements of the existing hydrogen in the structure.

Different tautomeric forms due to amide–iminol tautomerism were stable depending on the environment for paraxanthine, 1-methylxanthine, 7-methylxanthine, and xanthine. In the gas phase, the iminol form of the amide group in position 2 was found to be more stable, while in solution phases, the amide form was preferred. The energy differences between tautomeric forms with the lowest energies were smaller in the gas phase (1.8–2.2 kcal/mol) than in the solution phase (5–6 kcal/mol).

Interestingly, for theophylline, 1-methylxanthine, and xanthine, the energy differences in the gas phase between the lowest-energy N- and O-protonated forms are relatively small (1.7, 2.7, and 1.5 kcal/mol, respectively). Nevertheless, the absence of fragments hinting toward O-protonation was observed for these compounds, suggesting that calculations (at least for isolated molecules) do not provide sufficient information to discriminate between the behavior of caffeine metabolites. However, the lower theoretical calculations indicated two possible protonation sides and amide–iminol tautomerism to be major structural differences between the observed structures (see the SI for further discussion).

### Evaluating Gas-Phase Equilibrium of Protomers

For five compounds out of nine xanthine derivatives, two separable species were observed in ATD; however, for only two compounds, theobromine and 3-methylxanthine, the CID/HRMS spectra for high- and low-mobility species showed significant differences. Therefore, it was of interest to find if any structural changes upon activation and equilibration of the high- and low-mobility species can be pinpointed.

For this purpose, we designed here a so-called two-dimensional ion mobility experiment. First, the protomers are separated in the first IM, followed by an isolation, storage (∼20 ms), and reinjection of the species to the IM. The reinjection could be done with a tunable activation voltage; here, we used the lowest possible voltage for reinjection to minimize any unwanted fragmentation. As a result, the IMS^2^ allows separating protomers as well as determining the ATD of the species formed from the protomers during storage (re-equilibration) and reinjection.

**Figure 4:**
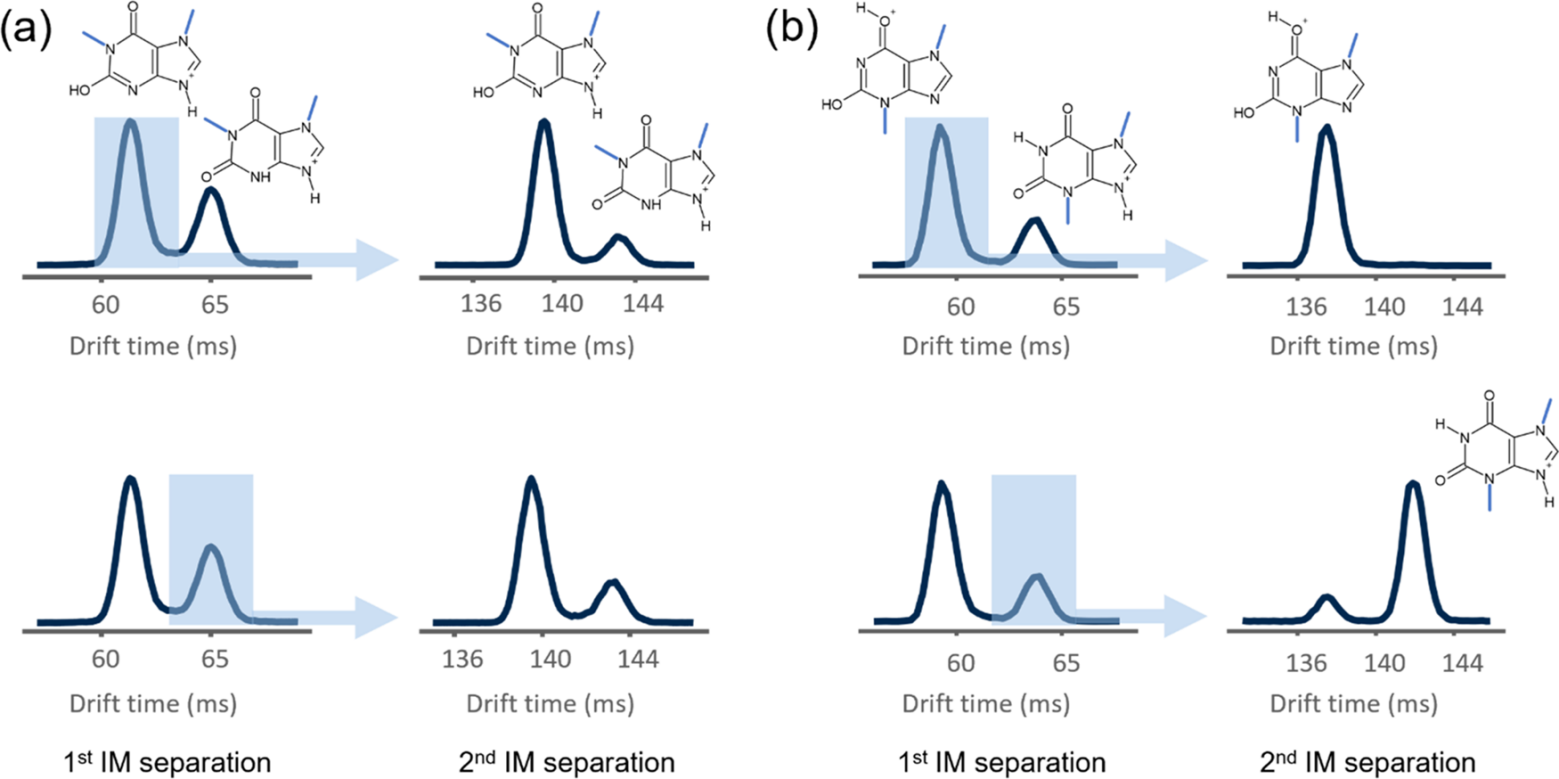
Two tautomers of paraxanthine (a) and theobromine (b) cations were separated with three cycles; one species was selected (blue rectangle) to reinject for the second ion mobility measurement. Using the same separation parameters for the second IM separation, two species were separated for paraxanthine. Although the first and second IM separation last the same length of time, the time scale is shifted for second separation as an additional time for reinjection is counted in when acquiring arrival time distributions. The rapid conversion was observed for paraxanthine, 1-methylxanthine, and 7-methylxanthine, while separation of one species was successful for theobromine and 3-methylxanthine. Calculated CCS values for respective structures are presented in Table 2.

Unexpectedly, reinjection of the high-mobility protomer of paraxanthine yielded two separable species in the ATD with the *m*/*z* of the parent ion. The ratio of the peaks was analogous to paraxanthine protomer peaks observed in the first ion mobility separation. Similarly, isolating and reinjecting low-mobility species resulted in two peaks in second ATD with a similar peak ratio (see Figure 4). The same effect was observed for 1-methylxanthine and 7-methylxanthine. A negative control experiment with theobromine resulted in the successful isolation of both high- and low-mobility species, and reinjection of the isolated peak resulted in one peak in second ion mobility separation, confirming the experiment workflow. Observation of two separable structural forms of paraxanthine after isolation and reinjection evidences the equilibrium between two converting structural forms. Additional calculations to determine collision cross sections (CCSs) confirmed the observed trend based on protomers of theobromine: the O-protonated structure had a smaller calculated collision cross section than the N-protonated molecule (see Table 2). Based on computations, the energy differences between amide–iminol tautomeric forms of paraxanthine, 1-methylxanthine, and 7-methylxanthine are relatively small in the gas phase (1.8–2.2 kcal/mol). This could explain the observation of two tautomers being in fast equilibrium (see Tables S4.1.–4.8 in the SI). Two-dimensional ion mobility experiments were conducted for all caffeine metabolites (see the SI).

**Table 2:**
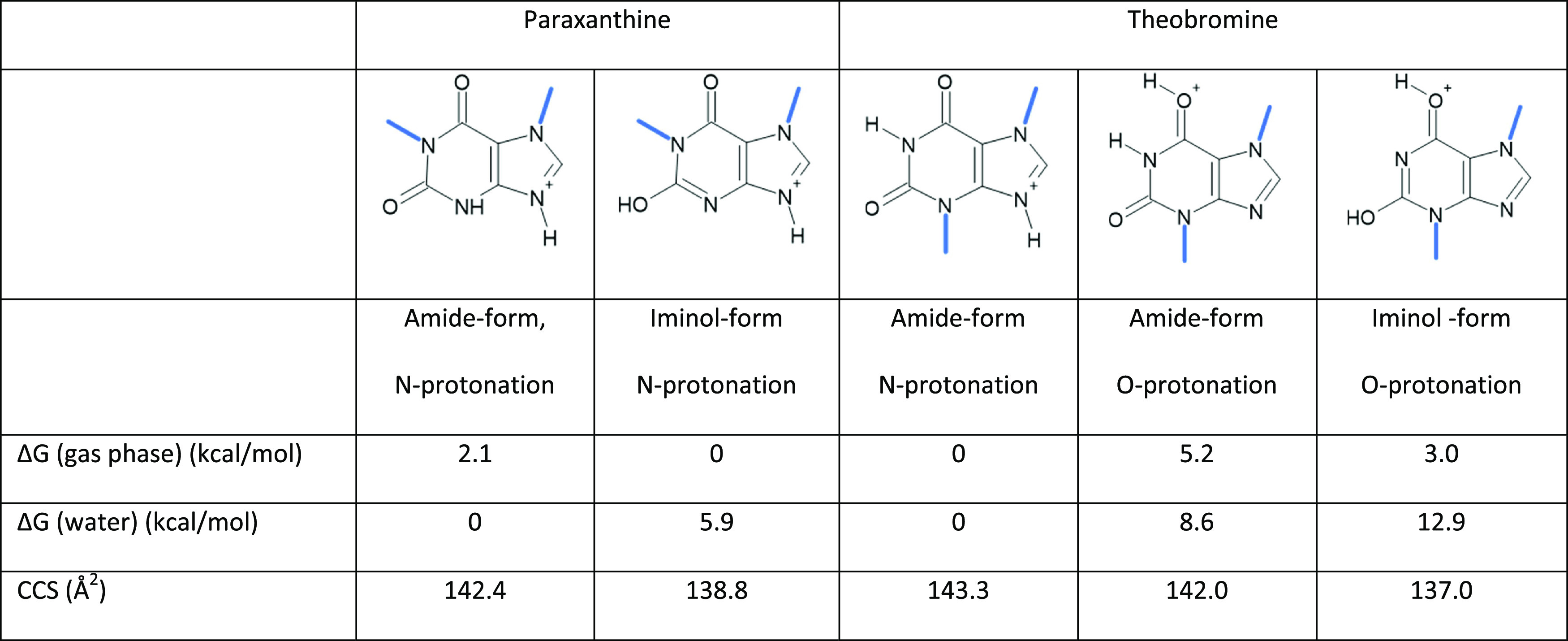
Calculated Gibbs Free Energies and Collision Cross Sections (CCS) of Most Stable Forms in the Gas Phase and the Solution Phase (Water)^a^

a Computational results in acetonitrile and water/acetonitrile mixture can be found in the SI. Energies are normalized for each compound by the lowest-energy tautomer in the gas or solution phase, respectively.

It can be seen from IMS^2^ experiments that two structures can interconvert in the gas phase only if the interconversion is intramolecular. For example, in the case of paraxanthine, the tautomers are kinetically interconvertible in the gas phase, resulting in two peaks in the ATD of IMS^2^ experiments. However, for theobromine, the deprotonation–protonation mechanism is needed for conversion between the high- and low-mobility species as the proton cannot move from the imidazole to the amide. Such a deprotonation–protonation mechanism is only possible in the presence of protic solvent molecules and not in the gas phase, and therefore a single peak is observed in the ATD of IMS^2^ experiments.

In the previously published literature on protomer formation, the most basic site in polyfunctional compounds containing amino and carboxyl groups can be different in the solution and gas phases and has been associated with the protomer formation.^5,10,13−15^ Differences between calculations and experimental results have been speculated to be caused by proton trapping with aprotic solvent molecules. The use of protic solvent has been reported to act as a proton carrier through different proposed mechanisms, such as water bridge,^10,41,42^ Grotthuss mechanism, and vehicle mechanism,^43^ and has been hypothesized to enable conversion of the molecular ion into an energetically more stable protomer in the gas phase. In this study, two caffeine metabolites, theobromine and 3-methylxanthine, behave similarly and suggest O-protonation when water is used as a solvent. However, calculations suggest one most basic site for all xanthine metabolites in both the solvent and gas phases (see Tables S4.1–4.8 in the SI). Therefore, forming a water bridge to relocate proton to produce a more stable protomer is unlikely. The reason might still lie in analyte–solvent clusters as solvent molecules can either bind to sites and block/inhibit protonation or form clusters with protonated sites. The kinetics of proton binding to different sites can also play a role, especially in case solvent molecules can trap proton to the faster binding site.

### Structural Fragments Indicating Protomer Formation

**Figure 5:**
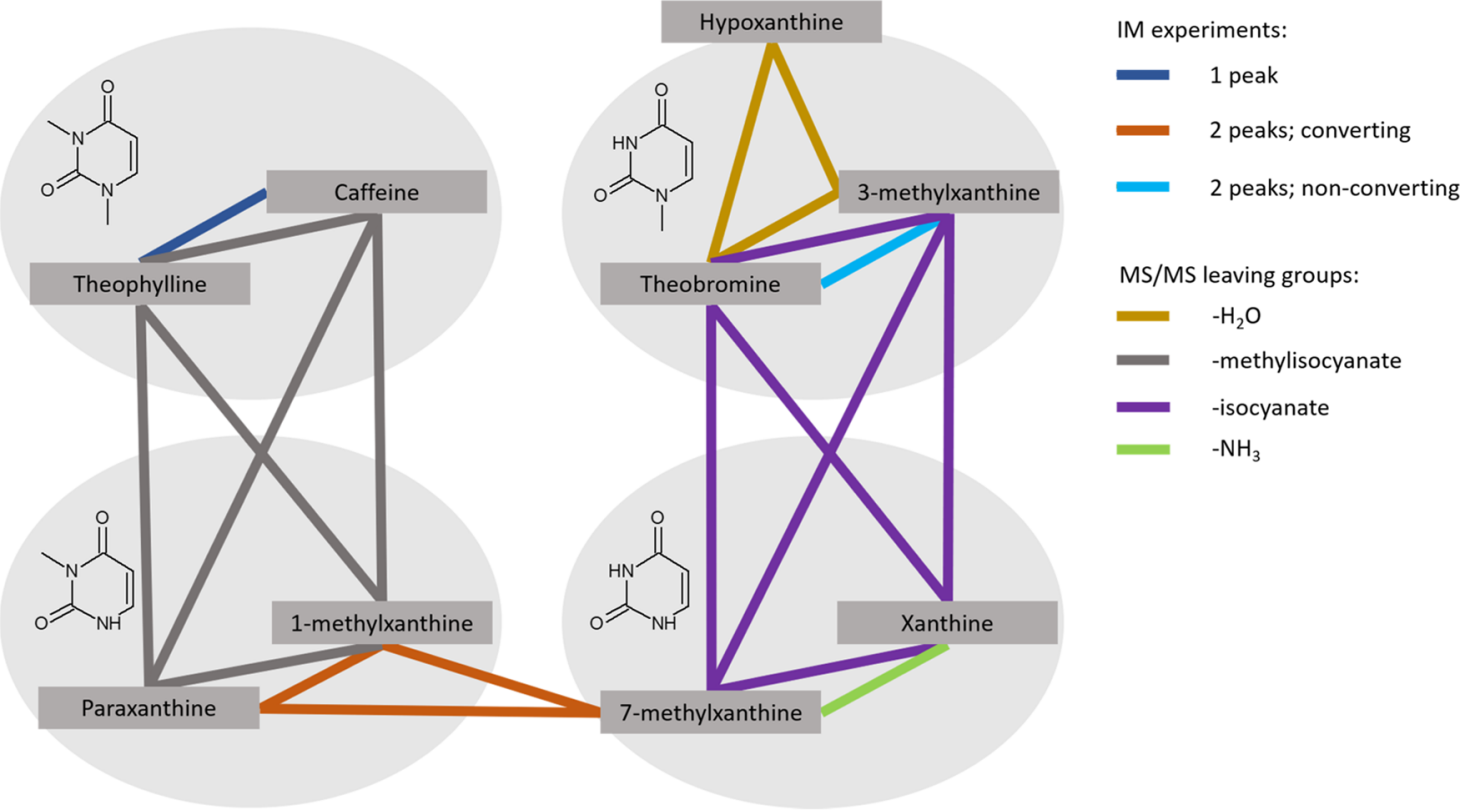
Structural fragments of methyl-substituted pyrimidine (without an imidazole ring), indicating similar behavior in protomer and tautomer formation.

Some structural parts seem to facilitate protomer formation. Behavior in the gas phase is mainly dictated by methyl-substituted pyrimidine, and compounds can be divided into groups based on these structural fragments (see Figure 5). First, the absence of the methyl group in position 3, which inhibits amide–iminol tautomerism for the amide in position 2, seems to play a key role in tautomer conversion based on paraxanthine, 1-methylxanthine, and 7-methylxanthine. Due to the low ionization efficiency of xanthine, similar behavior could not be confirmed.

The presence of a methyl group in position 3 and the absence of a methyl group in position 1 allowing stabilization by amide–iminol tautomerism of the amide group in position 6 is important to produce protomers with different fragmentation spectra, which was observed for theobromine and 3-methylxanthine. Hypoxanthine that is missing a carbonyl group in position 2 cannot form a tautomer with the nitrogen atom in position 3 and therefore behaves as theobromine and 3-methylxanthine, where tautomer formation is inhibited due to the methyl group. However, unlike for theobromine and 3-methylxanthine, the first peak of hypoxanthine was relatively small and no solvent effect was observed.

Based on 7-methylxanthine, the absence of both methyl groups seems to produce fast interconvertible tautomers, indicating that tautomer formation dominates over protomer formation, and protomers can only be formed when carbonyl groups are isolated from the imidazole ring with a methyl group in position 3. The presence of methyl groups in both positions 1 and 3 inhibits both protomer and tautomer formation, which was confirmed with observing one peak in IM for theophylline and caffeine. As a result, the combination of MS/MS, IM of the protomers, and IMS^2^ investigation of the tautomerism enables us to identify the location of the methyl groups in the xanthine scaffold for different caffeine metabolites (see Figure 5).

We conclude that protomer detection with IM and detection of tautomerism with IMS^2^ allows new possibilities in the structural elucidation of metabolic compounds. On the example of caffeine and its metabolites, we see that protomer and tautomer formation can be related to specific structural fragments. For example, theophylline and paraxanthine investigated here cannot be distinguished based on any other currently available spectral detail. However, for automated metabolome-wide application, additional developments will be required. First, the significance of protomer formation for other chemical classes of metabolites needs to be identified. This could capture a wide screening strategy resulting in a database. Second, the current automated data processing tools for automated untargeted metabolomics are rarely compatible with IM and protomer formation complicates the data processing further, essentially indicating two independent species. The tools today do not allow automatic processing of IMS^2^ spectra. Therefore, currently, the techniques proposed here are not yet high throughput and can be used for resolving the structure of selected features.

## Conclusions

We investigated protomer formation on the example of caffeine and its metabolites as well as nucleic bases and uric acid metabolites with similar structures. We found that the results of the two-dimensional ion-mobility experiments could be linked to the structure of the compound and provide information about proton hopping between different electronegative atoms in the caffeine metabolites. The solvent effect (water vs acetonitrile) observed in ion-mobility experiments confirmed the possibility of protomer formation. Therefore, gathering structure-dependent information from ion-mobility experiments could facilitate the identification of isomeric compounds.

## Supporting Information Available

Details of additionally measured structurally similar compounds and their arrival time distributions in water and acetonitrile; MS/MS spectra for caffeine and its metabolites (for each IM peak separately); two-dimensional ion-mobility experiments of caffeine metabolites; computational data for caffeine metabolites; identification with SIRIUS+FingerID software; ionization efficiency experiments and results; and raw data of ion mobility, fragmentation experiments, DFT-calculated structures, and their Gibbs free energies in all environments have been uploaded to Zenodo and can be accessed at https://doi.org/10.5281/zenodo.6637393 (PDF)
