# Supplementary material for: Protomer Formation Can Aid the Structural Identification of Caffeine Metabolites
Source: Anal Chem. 2022 Jul 21;94(30):10601–9. doi: 10.1021/acs.analchem.2c00257 (PMC9352149; doi:10.1021/acs.analchem.2c00257)
Supplement: Supplementary file 1 — ac2c00257_si_001.pdf [file ac2c00257_si_001.pdf]

Supplementary information for

Protomer formation can aid structural identification of caffeine metabolites

Helen Sepman,[a] Sofja Tshepelevitsh, [b] Henrik Hupatz [c] and Anneli Kruve [a*]

[a]Department of Materials and Environmental Chemistry, Stockholm University, Svante Arrhenius väg 16, 106 91 Stockholm, Sweden

[b]Institute of Chemistry, University of Tartu, Ravila 14a, Tartu 50411, Estonia

[c]Institut für Chemie und Biochemie, Freie Universität Berlin, Takustrasse 3, 14195 Berlin, Germany

ATDs measured in water and acetonitrile for all compounds and summary table for additional structurally similar compounds.

Table S1. Summary table for additional structurally similar compounds of xanthine metabolites.

Compound name	Structure	Number of IM peaks	Solvent effect
Guanine	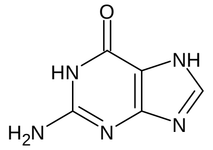	2	NO
Adenine	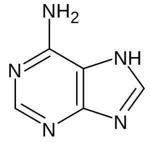	1*	-
Thymine	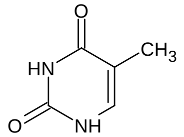	1*	-
1-methyluric acid	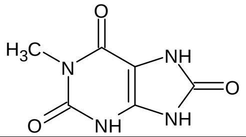	2	NO
1,3-dimethyluric acid	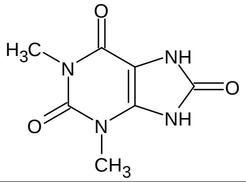	>2	YES
1,7-dimethyluric acid	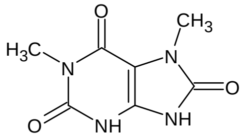	>2	YES
3,7-dimethyluric acid	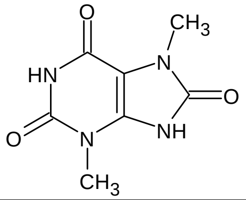	>2	YES
1,3,7,9,-tetramethyluric acid	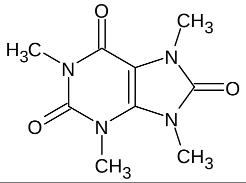	2	YES

* Number of peaks could not be identified with full confidence due to low ionization efficiency of either the species or substance.

Figure S1. ATD measured in water and acetonitrile for caffeine.

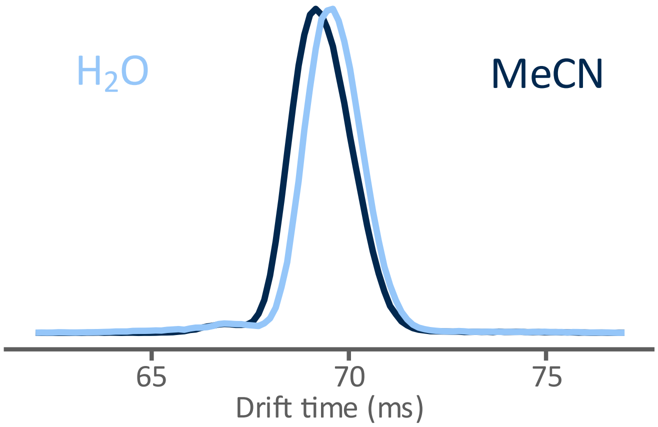

Figure S2. ATD measured in water and acetonitrile for theophylline.

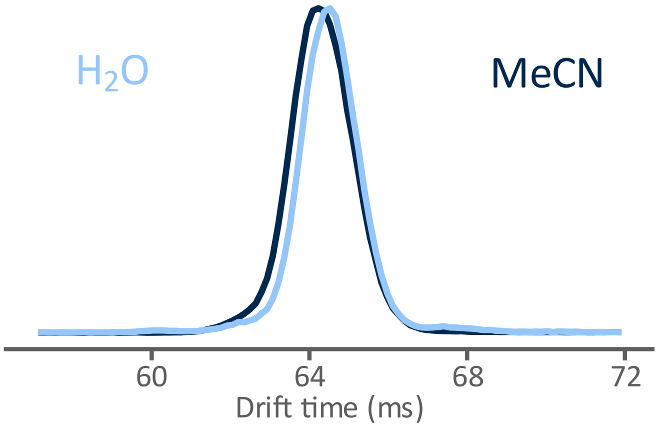

Figure S3. ATD measured in water and acetonitrile for paraxanthine.

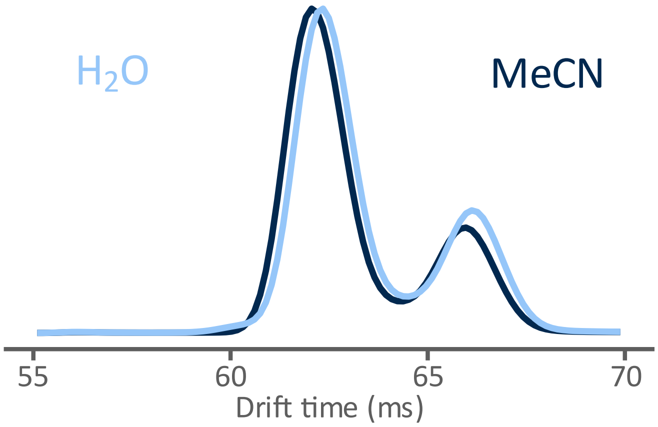

Figure S4. ATD measured in water and acetonitrile for theobromine.

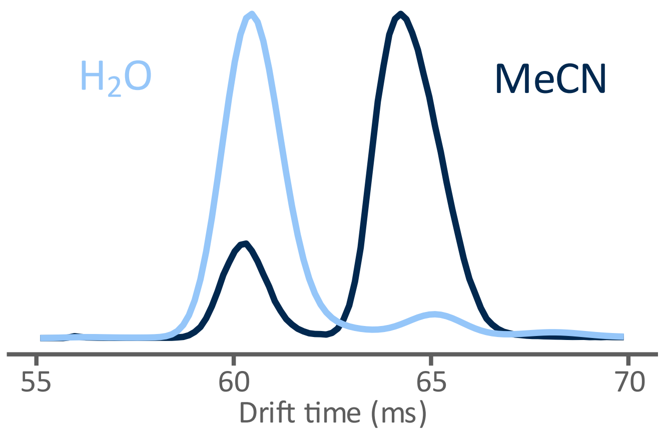

Figure S5. ATD measured in water and acetonitrile for 1-methylxanthine.

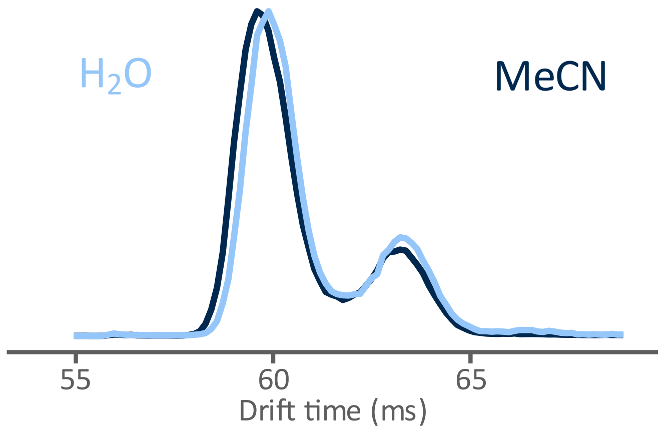

Figure S6. ATD measured in water and acetonitrile for 3-methylxanthine.

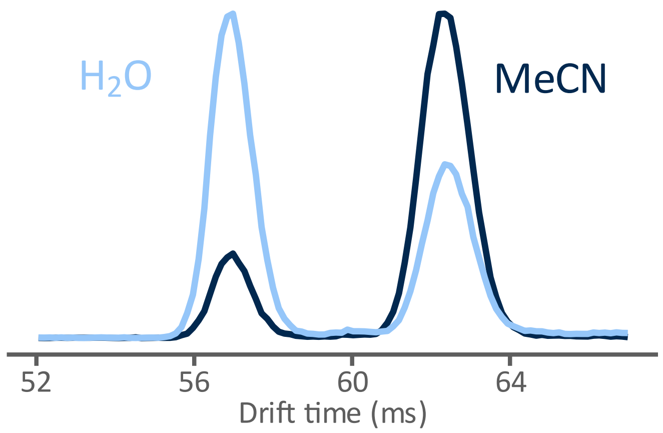

Figure S7. ATD measured in water and acetonitrile for 7-methylxanthine.

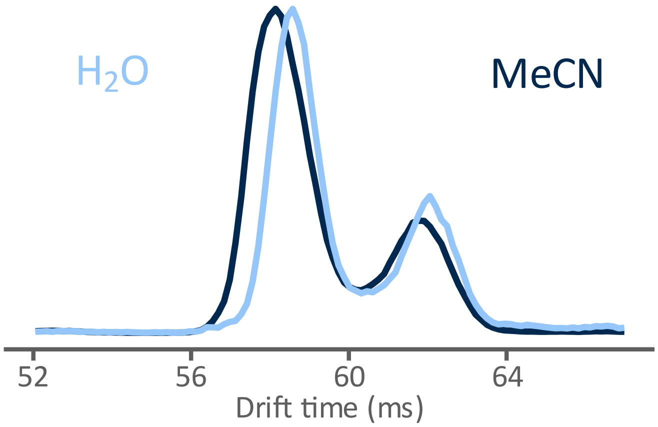

Figure S8. ATD measured in water and acetonitrile for xanthine.

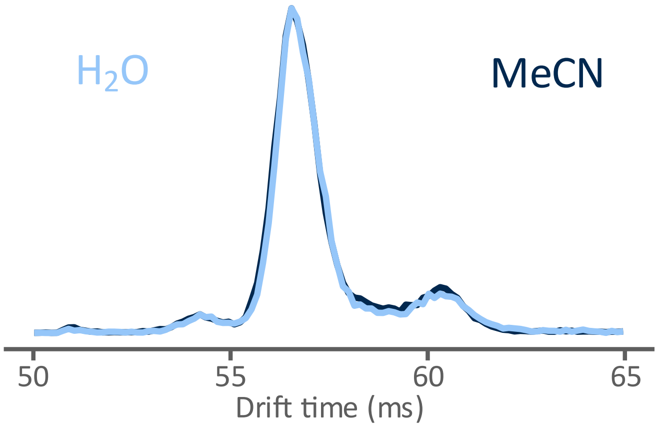

Figure S9. ATD measured in water and acetonitrile for hypoxanthine.

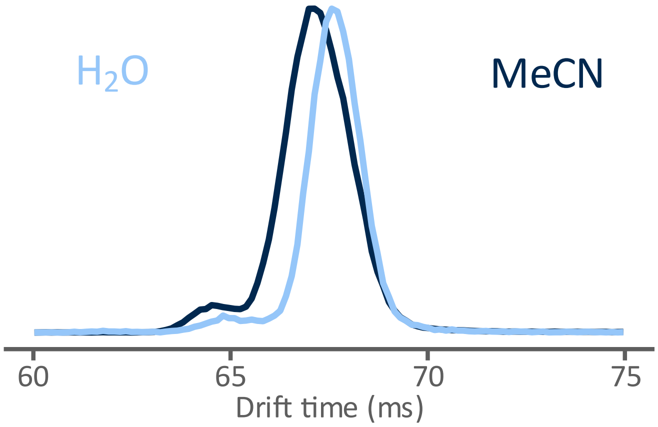

Figure S10. ATD measured in water and acetonitrile for guanine.

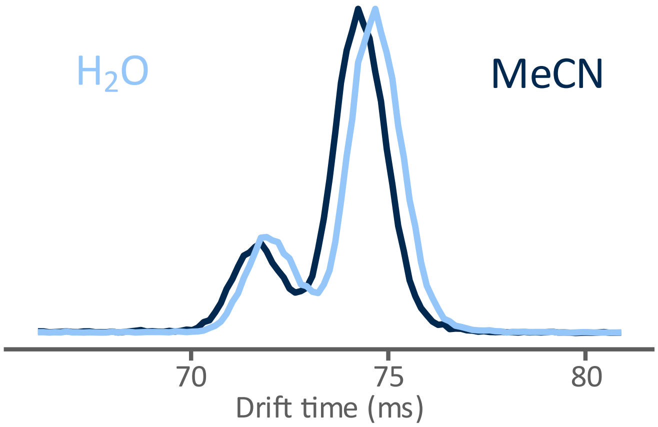

Figure S11. ATD measured in water and acetonitrile for adenine.

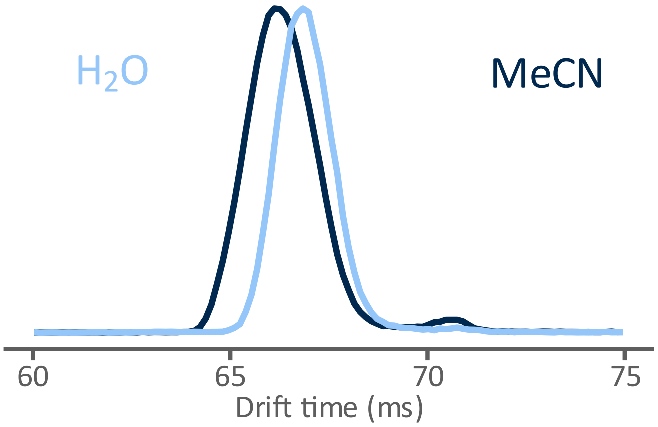

Figure S12. ATD measured in water and acetonitrile for thymine.

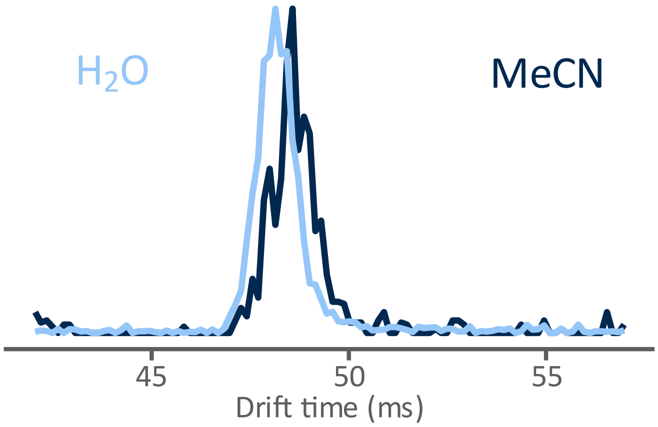

Figure S13. ATD measured in water and acetonitrile for 1-methyluric acid.

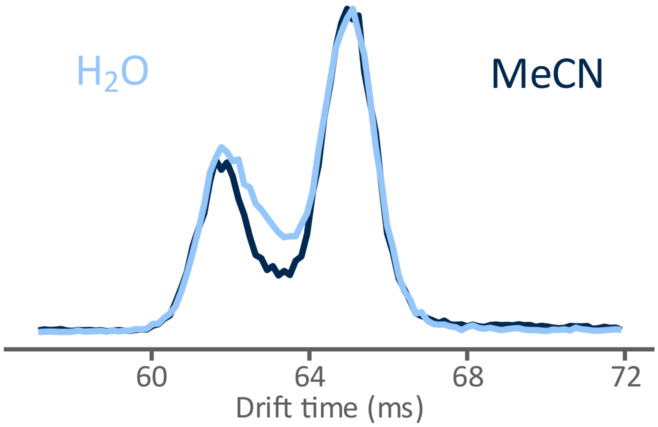

Figure S14. ATD measured in water and acetonitrile for 1,3-dimethyluric acid.

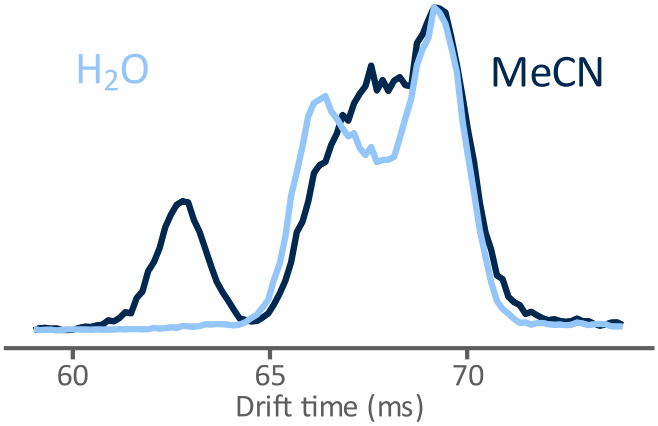

Figure S15. ATD measured in water and acetonitrile for 1,7-dimethyluric acid.

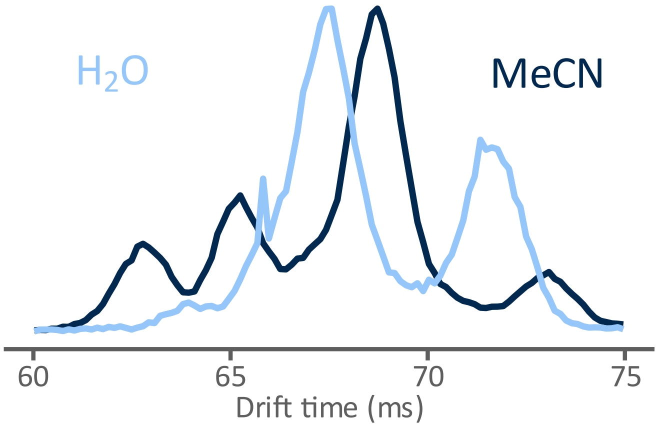

Figure S16. ATD measured in water and acetonitrile for 3,7-dimethyluric acid.

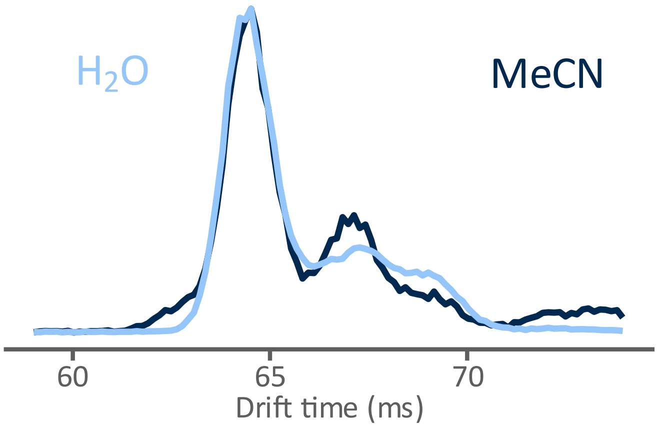

Figure S17. ATD measured in water and acetonitrile for 1,3,7,9,-tetramethyluric acid.

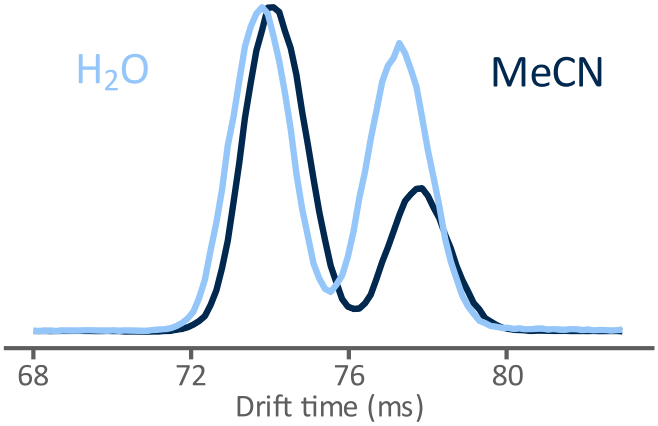

MS/MS spectra for all caffeine metabolites (for each IM separated peak) with collision energy of 20V.

Figure S18. MS/MS spectrum of caffeine.

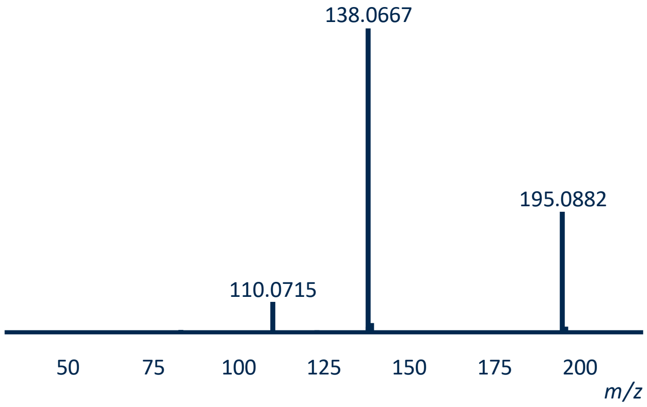

Figure S19. MS/MS spectrum of theophylline.

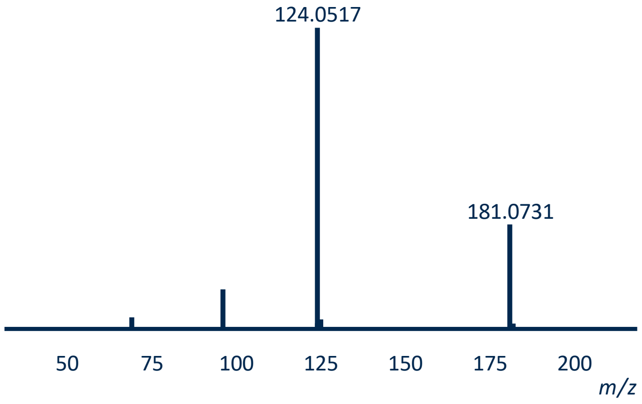

Figure S20. MS/MS spectrum of paraxanthine (high mobility species).

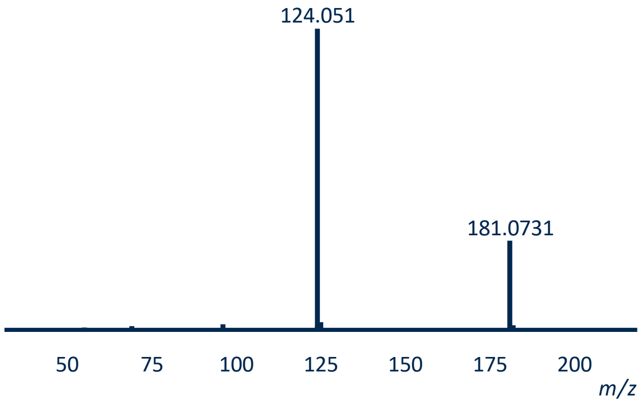

Figure S21. MS/MS spectrum of paraxanthine (low mobility species).

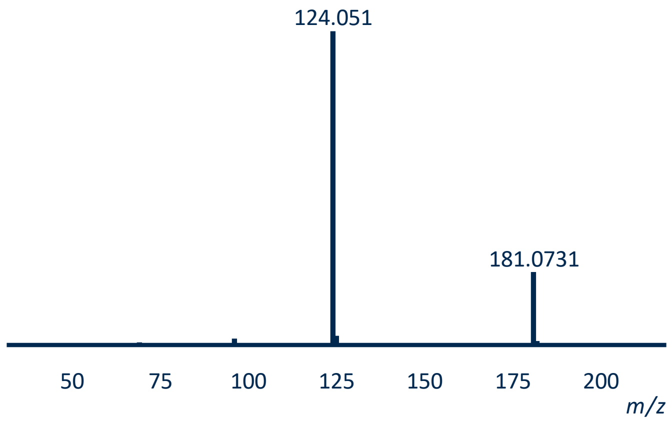

Figure S22. MS/MS spectrum of theobromine (high mobility species).

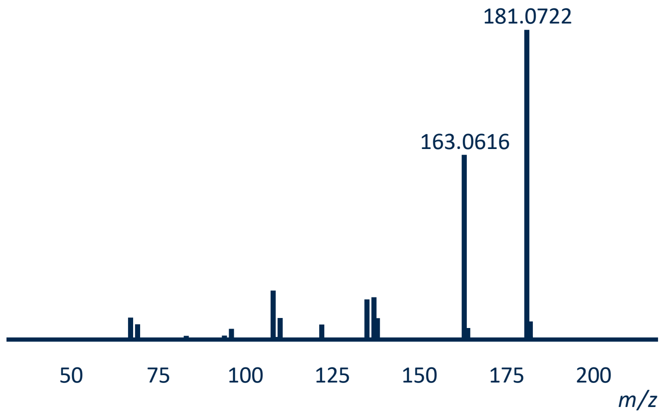

Figure S23. MS/MS spectrum of theobromine (low mobility species).

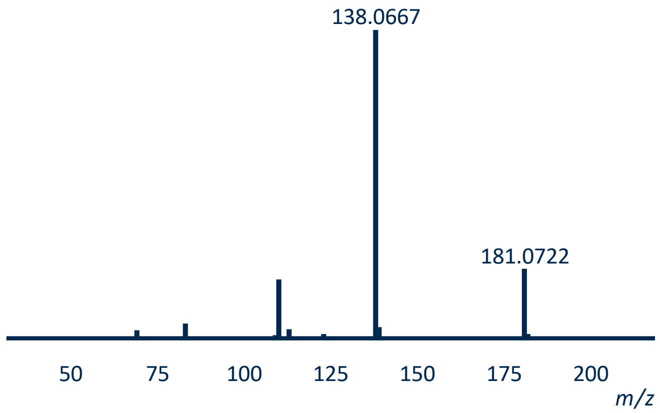

Figure S24. MS/MS spectrum of 1-methylxanthine (high mobility species).

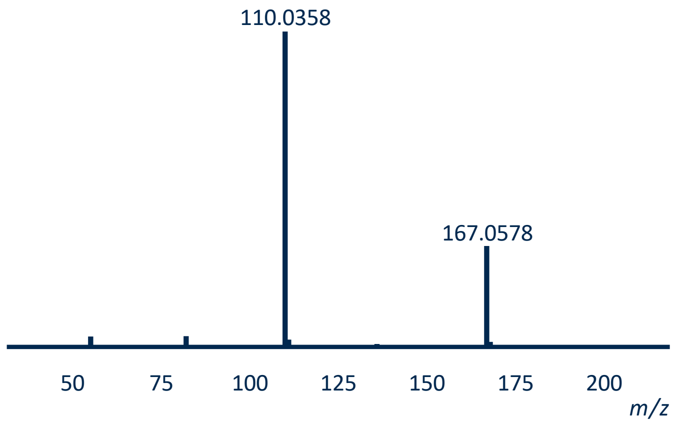

Figure S25. MS/MS spectrum of 1-methylxanthine (low mobility species).

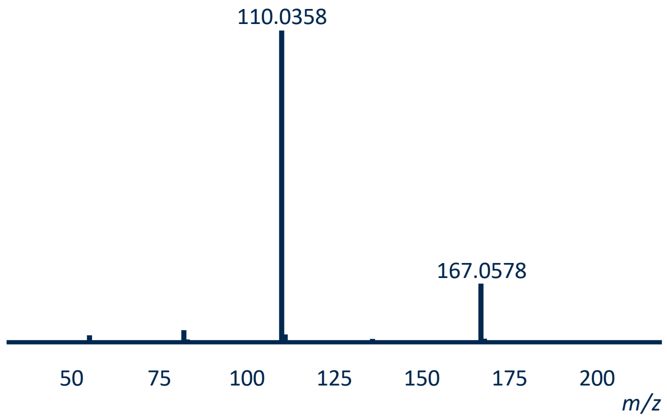

Figure S26. MS/MS spectrum of 3-methylxanthine (high mobility species).

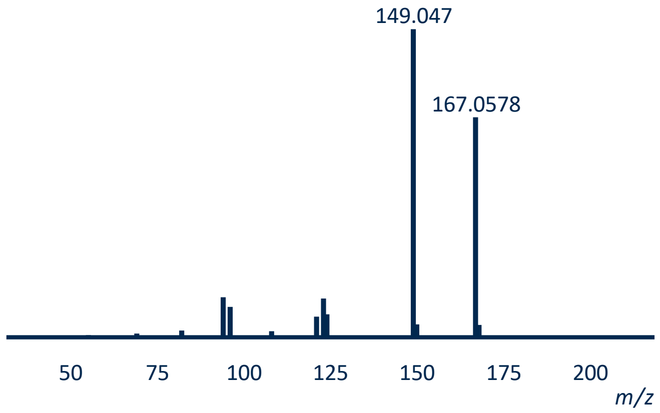

Figure S27. MS/MS spectrum of 3-methylxanthine (low mobility species).

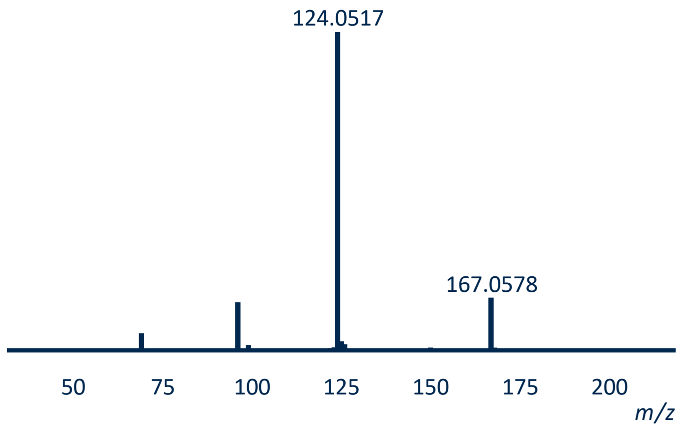

Figure S28. MS/MS spectrum of 7-methylxanthine (high mobility species).

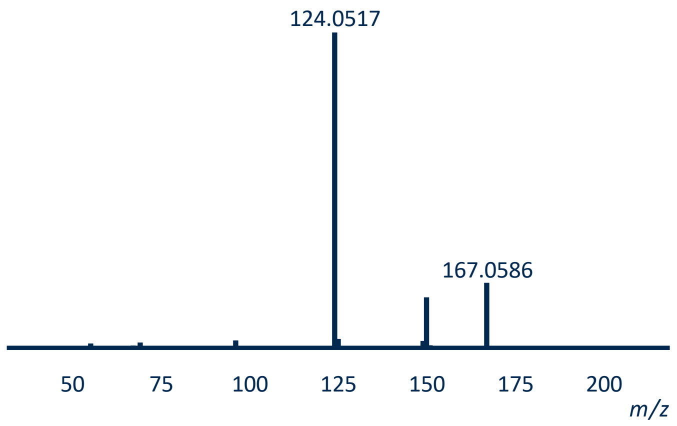

Figure S29. MS/MS spectrum of 7-methylxanthine (low mobility species).

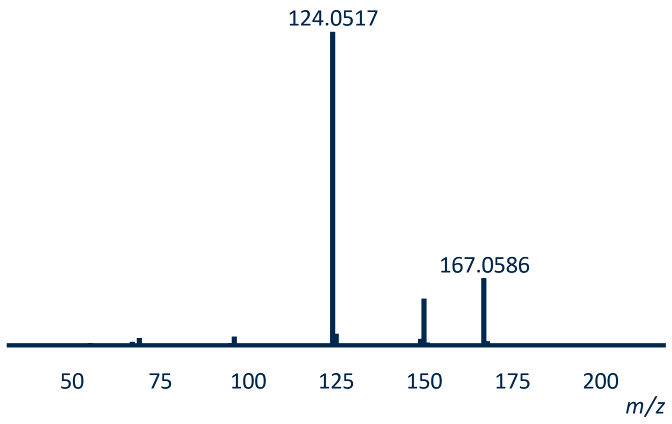

Figure S30. MS/MS spectrum of xanthine.

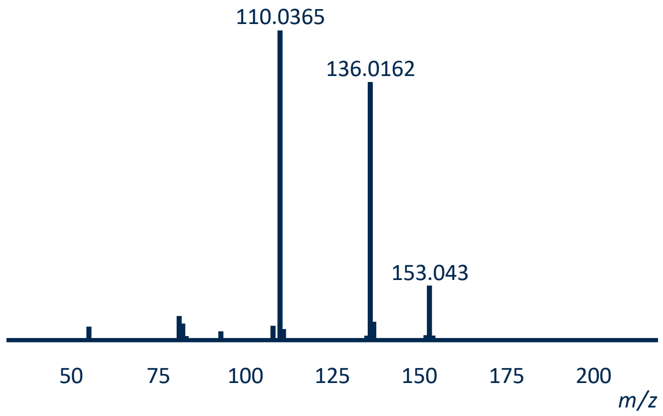

Two-dimensional ion mobility experiments (IMS^2)

To carry out IMS^2 experiments, separation with three cycles was performed with cyclic ion mobility. Then, one mobility species was selected and reinjected as well as another separation using three cycles was performed.

Figure S31. ATD of high mobility species of paraxanthine for second ion mobility separation.

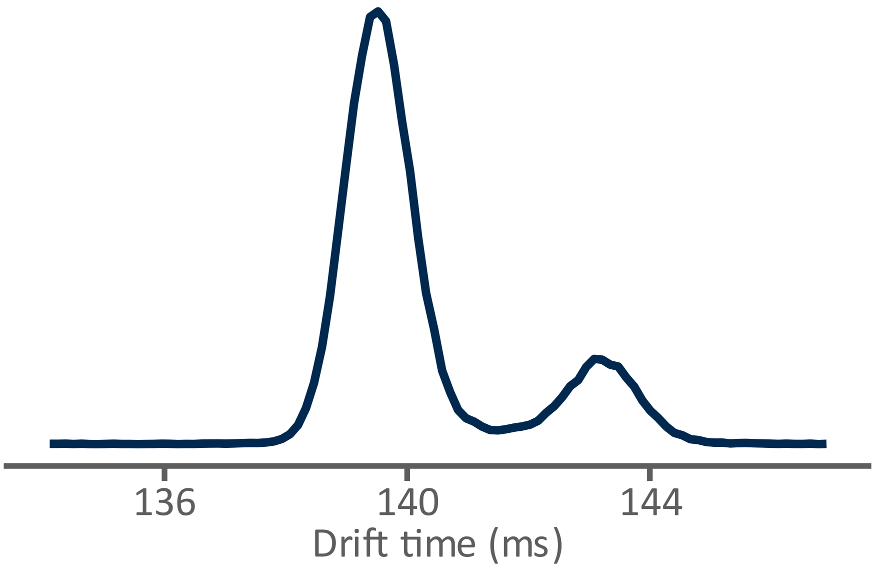

Figure S32. ATD of low mobility species of paraxanthine for second ion mobility separation.

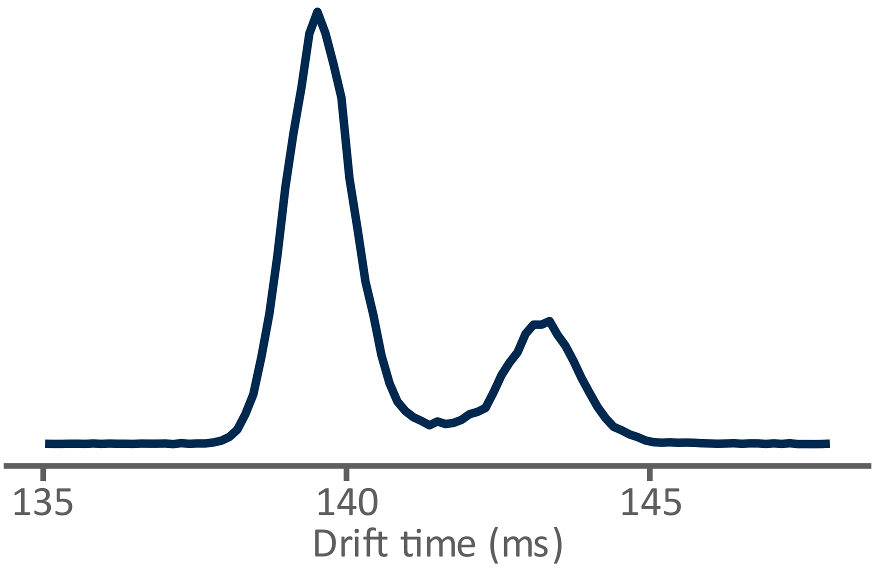

Figure S33. ATD of high mobility species of theobromine for second ion mobility separation.

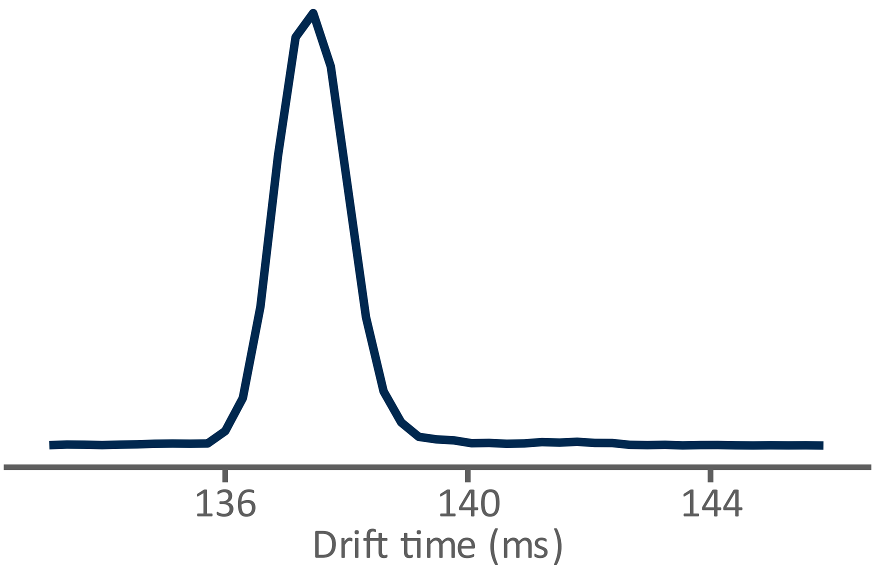

Figure S34. ATD of low mobility species of theobromine for second ion mobility separation.

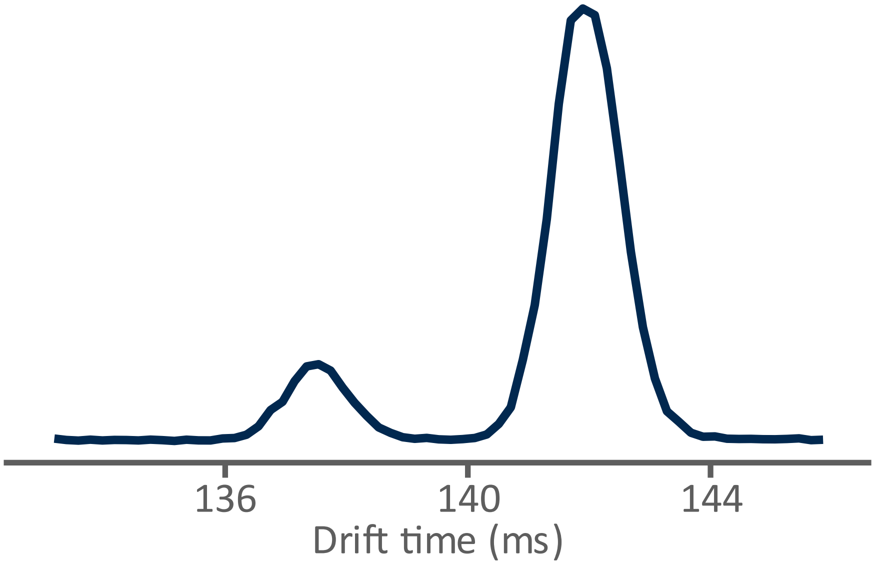

Figure S35. ATD of high mobility species of 1-methylxanthine for second ion mobility separation.

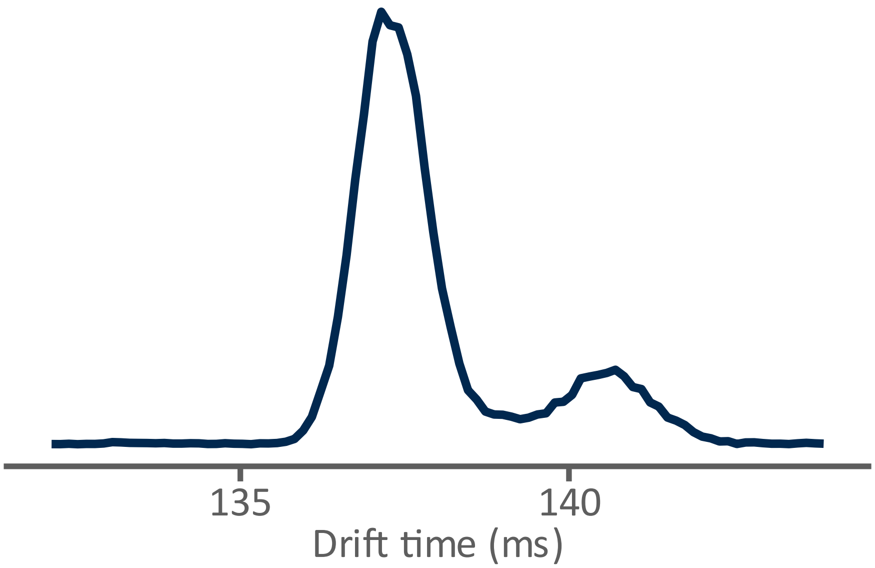

Figure S36. ATD of low mobility species of 1-methylxanthine for second ion mobility separation.

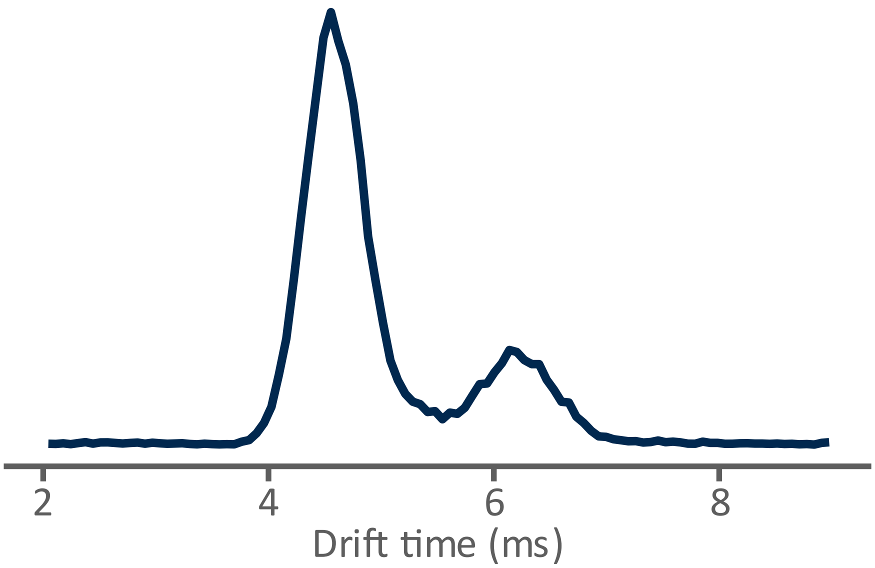

Figure S37. ATD of high mobility species of 3-methylxanthine for second ion mobility separation.

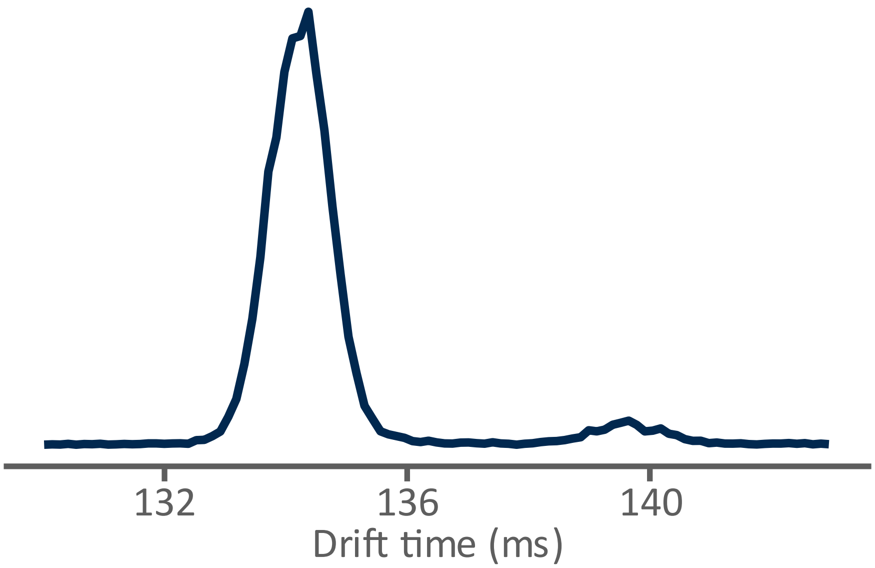

Figure S38. ATD of low mobility species of 3-methylxanthine for second ion mobility separation.

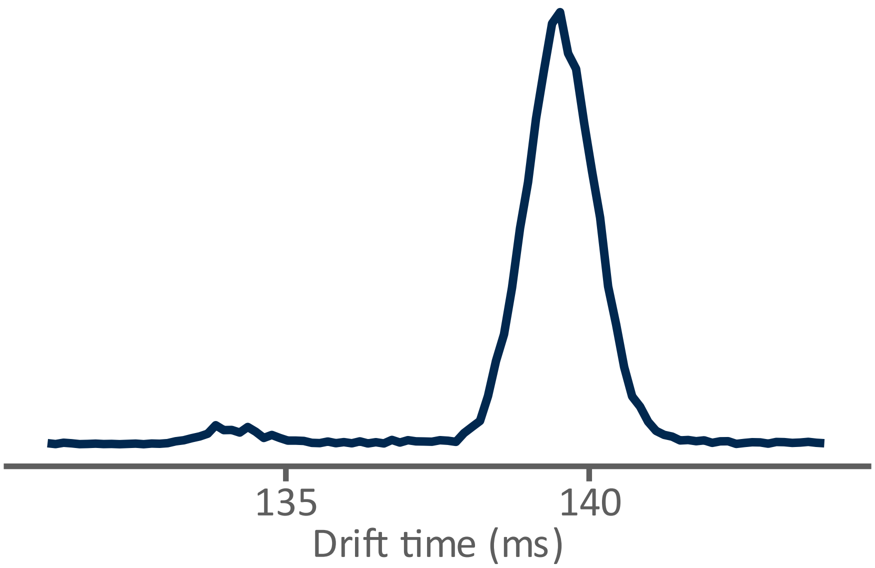

Figure S39. ATD of high mobility species of 7-methylxanthine for second ion mobility separation.

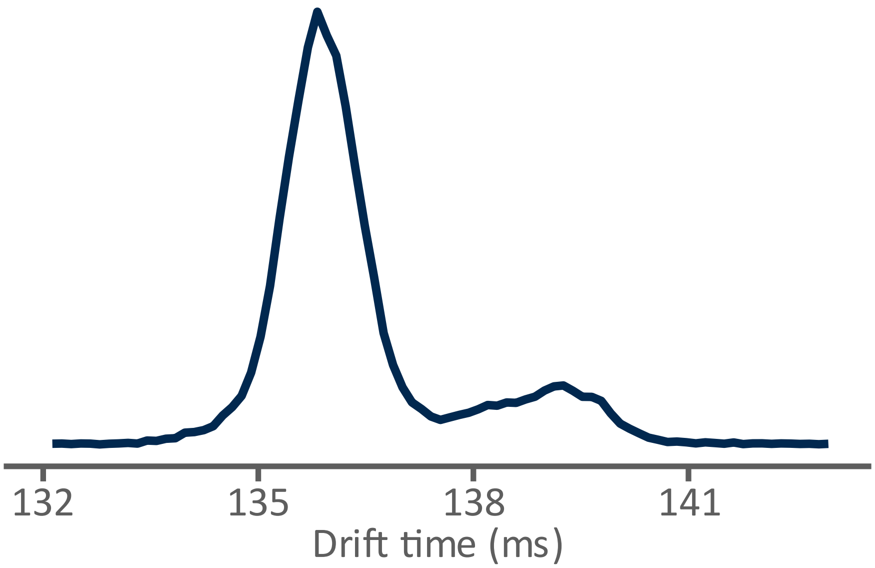

Figure S40. ATD of high mobility species of 7-methylxanthine for second ion mobility separation.

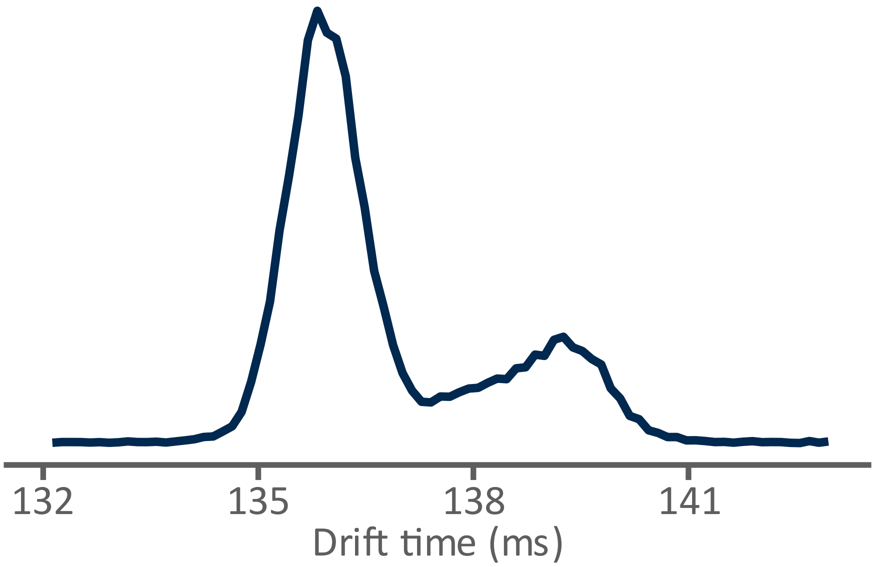

Computational data

COSMO-RS[1–3] (Conductor-Like Screening Model for Real Solvents) method is widely used for modeling fluid mixtures.[4,5] Its unrivalled advantage over other methods is its ability to model arbitrary multicomponent mixtures without additional solvent-specific parametrization and at a very low computational cost. The method consists of two steps. The first is geometry optimization of all involved compounds (solvents and solutes) in ideal conductor using Density Functional Theory, optionally followed by single-point calculation with larger basis set. It yields the energy of the molecule embedded in virtual conductor, and charge distribution on its surface. The second step is statistical thermodynamic procedure, where interaction energies of species in solution are computed from pair-wise interactions of molecular surface segments. Total energies of species in mixtures are combined from their energies in conductor and interaction energies with other components.

Intramolecular hydrogen bonds are taken into account in the first step: their stabilizing effect is reflected in the energy of the particle in conductor. Intermolecular hydrogen bonds are accounted for in the second step via polarization charge densities of separated donor and acceptor atoms in virtual conductor.[6,7] These quantities were found to be well correlated with the energies of the resulting hydrogen bonds.[6,7] Hydrogen bond formation is expected when the charge densities on the involved atoms exceed certain thresholds.

The calculation results for neutral and protonated structures were compared with the available literature data. Our calculations aligned with literature values for structures of neutral and protonated hypoxanthine in water.[8] Our conclusions for neutral hypoxanthine, theophylline and theobromine in the gas phase are in agreement with computational results from literature obtained using different computational methods.[9–13] Our results for xanthine in water are in semi-quantitative agreement with calculated data.[13] Experimental evidence from Lichtenberg et al. validates the conclusions concerning neutral forms of unsubstituted and methylated xanthines in liquids.[14] On the other hand, the authors' suggestion that these compounds become protonated at carbonyl group contradicts our results that indicate that protonation happens almost exclusively at the imidazole nitrogen. Our computations do not fully agree with the neutral gas phase structures reported for hypoxanthine and xanthine.[15,16] The applied computational method was demonstrated to be very reliable in identifying the most stable conformers in liquid phases, although their quantitative abundances were reproduced less accurately and the accuracy in case of charged species could be lower still. [17]

Table S2. Gibbs free energies of protonated caffeine. Energies are in kcal mol^{-1} and normalized to lowest energy form in gas phase and solution phase, respectively.

Structure	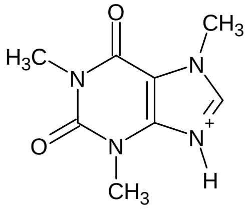	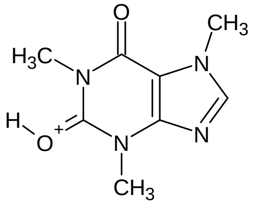	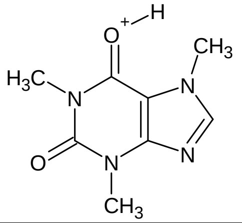
gas	0	6.7	5.7
H_2O	1.5	14.4	12.6
H_2O/MeCN 20/80 (v/v)	0.3	12.6	11.0
MeCN	0	12.0	10.4

Table S3. Gibbs free energies of protonated theophylline. Energies are in kcal mol^{-1} and normalized to lowest energy form in gas phase and solution phase, respectively.

Structure	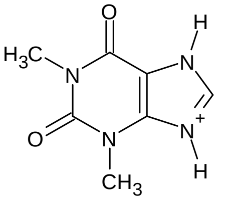	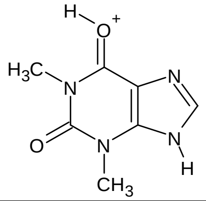	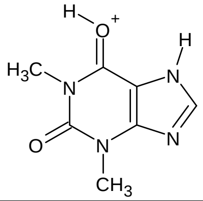
gas	0	1.7	4.8
H_2O	0.9	11.7	10.5
H_2O/MeCN 20/80 (v/v)	0	10.4	9.1
MeCN	0.8	10.6	9.6

Table S4. Gibbs free energies of protonated paraxanthine. Energies are in kcal mol^{-1} and normalized to lowest energy form in gas phase and solution phase, respectively.

Structure	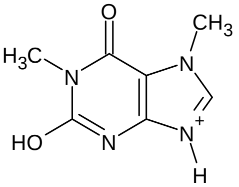	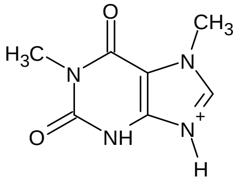
gas	0	2.0
H_2O	6.9	1.0
H_2O/MeCN 20/80 (v/v)	5.5	0
MeCN	5.7	0.4

Table S5. Gibbs free energies of protonated theobromine. Energies are in kcal mol^{-1} and normalized to lowest energy form in gas phase and solution phase, respectively.

Structure	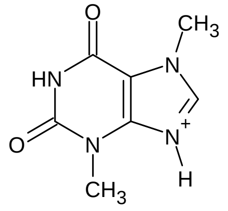	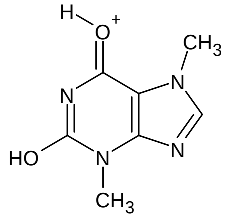	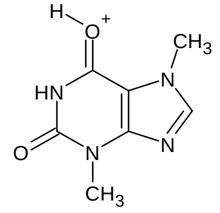
gas	0	3.0	7.2
H_2O	0.8	13.7	9.4
H_2O/MeCN 20/80 (v/v)	0	11.9	8.0
MeCN	0.3	12.0	8.7

Table S6. Gibbs free energies of protonated 1-methylxanthine. Energies are in kcal mol^{-1} and normalized to lowest energy form in gas phase and solution phase, respectively.

Structure	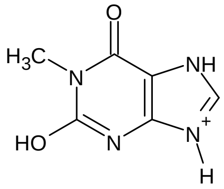	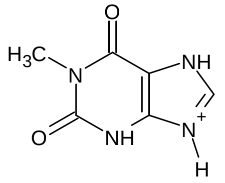	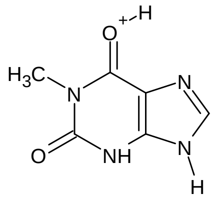
gas	0	1.8	4.5
H_2O	6.2	0.6	11.6
H_2O/MeCN 20/80 (v/v)	5.2	0	10.7
MeCN	6.6	1.5	11.8

Table S7. Gibbs free energies of protonated 3-methylxanthine. Energies are in kcal mol^{-1} and normalized to lowest energy form in gas phase and solution phase, respectively.

Structure	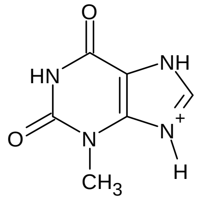	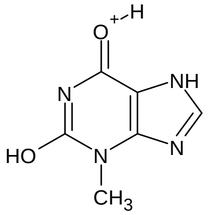	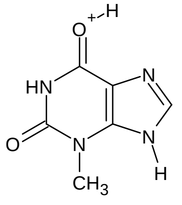	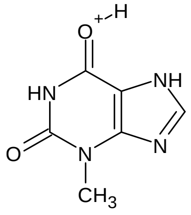
gas	0	0.5	2.7	4.5
H_2O	0.4	13.2	9.8	8.8
H_2O/MeCN 20/80 (v/v)	0	11.9	8.9	7.9
MeCN	1.4	12.8	10.2	9.4

Table S8. Gibbs free energies of protonated 7-methylxanthine. Energies are in kcal mol^{-1} and normalized to lowest energy form in gas phase and solution phase, respectively.

Structure	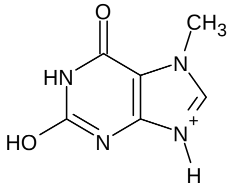	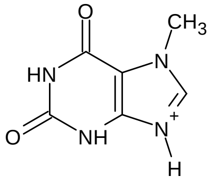	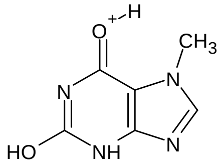
gas	0	1.9	5.9
H_2O	5.8	0.5	13.7
H_2O/MeCN 20/80 (v/v)	5.0	0	12.5
MeCN	6.0	1.0	13.7

Table S9. Gibbs free energies of protonated xanthine. Energies are in kcal mol^{-1} and normalized to lowest energy form in gas phase and solution phase, respectively.

Structure	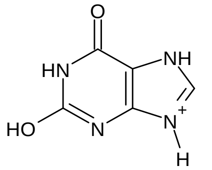	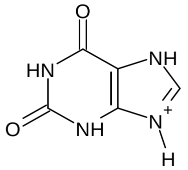	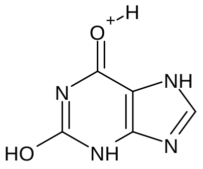	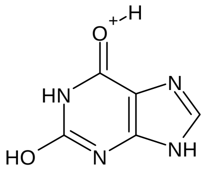
gas	0	2.2	3.7	6.7
H_2O	5.4	0.1	13.5	16.0
H_2O/MeCN 20/80 (v/v)	5.0	0	12.6	15.2
MeCN	7.1	2.1	14.7	17.3

Table S10. Gibbs free energies of protonated hypoxanthine. Energies are in kcal mol^{-1} and normalized to lowest energy form in gas phase and solution phase, respectively.

Structure	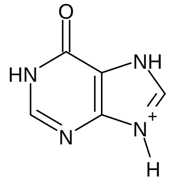	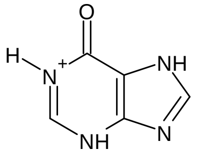	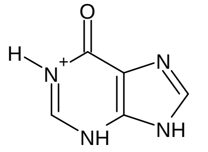
gas	0	2.5	13.6
H_2O	0.1	2.7	5.6
H_2O/MeCN 20/80 (v/v)	0	2.4	5.5
MeCN	1.5	3.9	7.4

Identification with SIRIUS+Finger:ID software

SIRIUS+Finger:ID[18–20] software was used to predict molecular formula and structural fingerprints based on predicted fragmentation tree from acquired MS/MS data. Possible molecular structures were proposed using CSI:FingerID web service for molecular databases, based on matching scores of fingerprints predicted from MS/MS spectra and fingerprints calculated for compounds in databases.

Generally, the formation of different species in CID depending on the dominating protomer can complicate the interpretation of the fragmentation spectra as a mixture of different fragmentation pathways is observed. To assess the extent of the effect that protomer formation has on the identification of caffeine metabolites, we used SIRIUS+Finger:ID software to predict molecular fingerprints based on the calculated fragmentation trees. The predicted fingerprints are later compared to fingerprints of compounds in databases and possible structures are proposed. The matches were separately calculated for both high- and low-mobility species of theobromine and paraxanthine measured in water/acetonitrile (20/80, v/v) mixture.

The molecular formula was correctly predicted with high confidence for both peaks of both substances based on computations, the respective Sirius scores were 100.0% and 99.9% for theobromine's high and low mobility species and 99.7% and 98.7% for paraxanthine's high and low mobility species. The probabilistic fingerprints for the high- and low-mobility species showed some differences, see Figure S5.1. We further investigated molecular fingerprints that were predicted for only one mobility species of the compound i.e. fingerprints that are predicted for one protomer/tautomer ($p > 0.5$) and not for the other ($p < 0.5$). However, these fingerprints were associated to amide-groups and heterocycles containing nitrogen and were generally descriptive for both mobility species. Therefore, predicted fingerprints did not carry protomer or tautomer specific information.

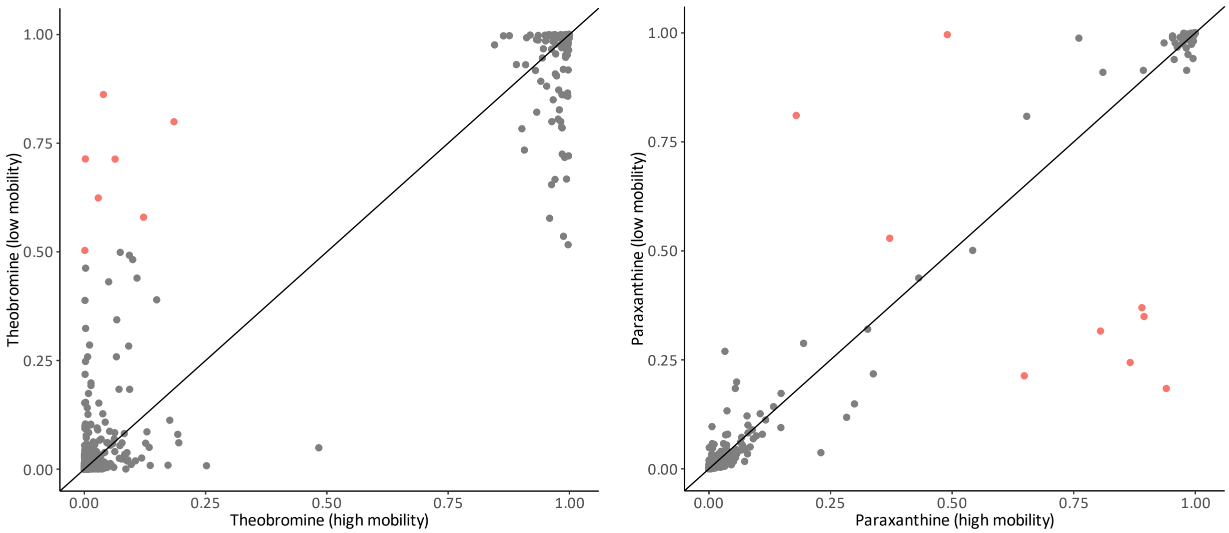

Figure S41. Correlations between predicted probabilities of molecular fingerprints present in high and low mobility species of a) theobromine and b) paraxanthine.

Fingerprint calculation and compound structure identification with SIRIUS+Finger:ID was also carried out for a combined MS/MS spectrum of high- and low-mobility species of theobromine and paraxanthine and for theophylline. For all compounds, the correct molecular formula was predicted with high confidence. The correct isomeric structure was predicted for theobromine and paraxanthine; however, for theophylline that has one mobility species, the correct isomeric structure was predicted as the fourth probable structure and paraxanthine was predicted as the first probable structure. For both theophylline and paraxanthine, the fragmentation spectra are identical as main fragment formed after loss of methylisocyanate has the same *m/z* value, which complicates the identification of these isomers. However, information gathered from ion mobility experiments would help to differentiate between these compounds as paraxanthine has two separable structural forms which is not true for theophylline.

Table S11. Fingerprints predicted for low mobility species ($p_{low} > 0.5$) but not for high mobility species ($p_{high} < 0.5$) of theobromine.

absoluteIndex	p_{high}	p_{low}	Structural unit
900	0.04	0.86	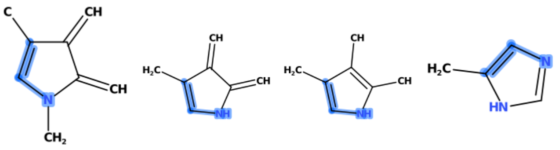
1067	0.12	0.58	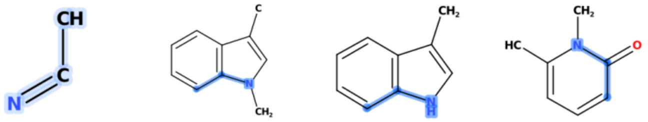
1184	0.18	0.80	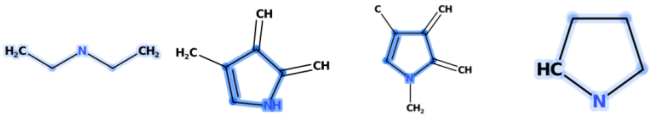
6316	0.06	0.71	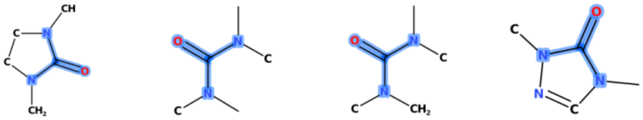
6324	<0.01	0.71	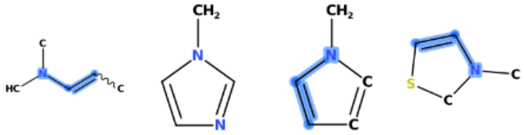
6366	0.02	0.62	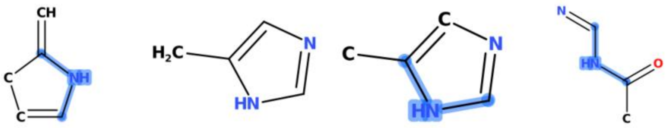
7533	<0.01	0.50	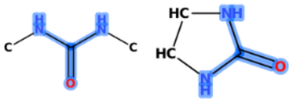

Table S12. Fingerprints predicted for high mobility species (p_{high} > 0.5) but not for low mobility species (p_{low} < 0.5) of paraxanthine.

absoluteIndex	p_{high}	p_{low}	Structural unit
233	0.89	0.35	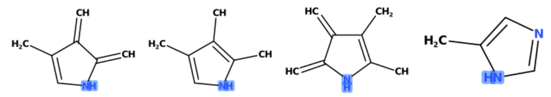
928	0.89	0.37	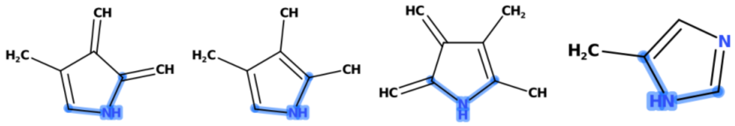
1016	0.87	0.24	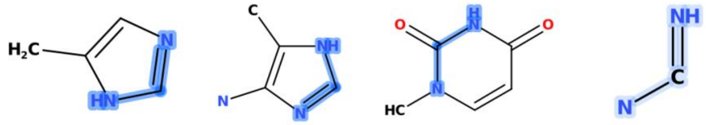
5792	0.94	0.18	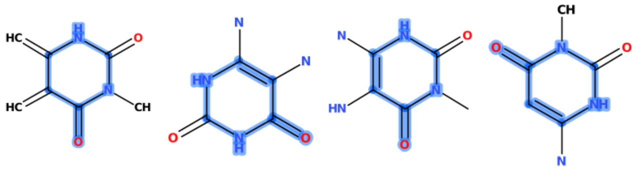
8077	0.65	0.21	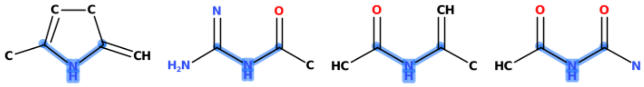
8171	0.81	0.31	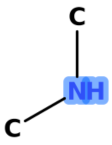

Table S13. Fingerprints predicted for low mobility species ($p_{low} > 0.5$) but not for high mobility species ($p_{high} < 0.5$) of paraxanthine.

absoluteIndex	p_{high}	p_{low}	Structural unit
461	0.37	0.53	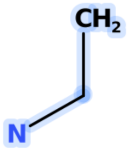 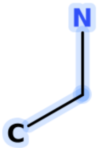
6316	0.49	0.99	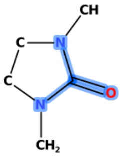 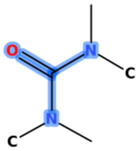 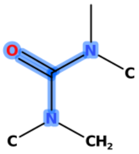 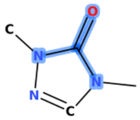
7546	0.18	0.81	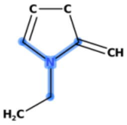 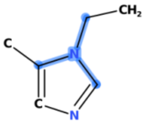 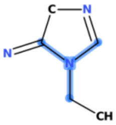 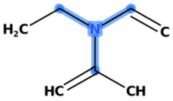

Table S14 First three predicted molecular formulas for paraxanthine high mobility species in water/acetonitrile (20/80).

Rank	Molecular formula	Adduct	Sirius score (%)	Tree score
1	$C_7H_8N_4O_2$	[M + H]+	99.650	18.90647
2	$C_9H_{10}NO_3$	[M + H]+	0.114	12.13515
3	$C_6H_{13}ClN_2O_2$	[M + H]+	0.099	11.99707

Table S15. First five predicted structures for molecular formula $C_7H_8N_4O_2$ (correct structure is marked with green) for paraxanthine high mobility species in water/acetonitrile (20/80).

Rank	Structure	CSI:FingerIDScore
1	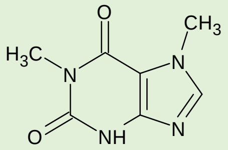	-9.953535
2	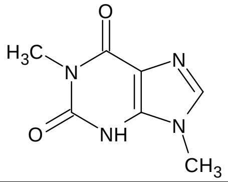	-28.532483
3	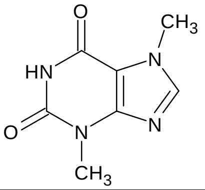	-31.905834
4	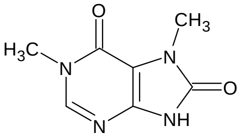	-50.061907
5	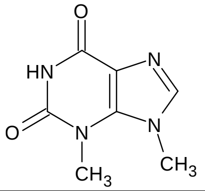	-51.947757

Figure S42. Fragmentation tree of molecular formula $C_7H_8N_4O_2$ for paraxanthine high mobility species in water/acetonitrile (20/80).

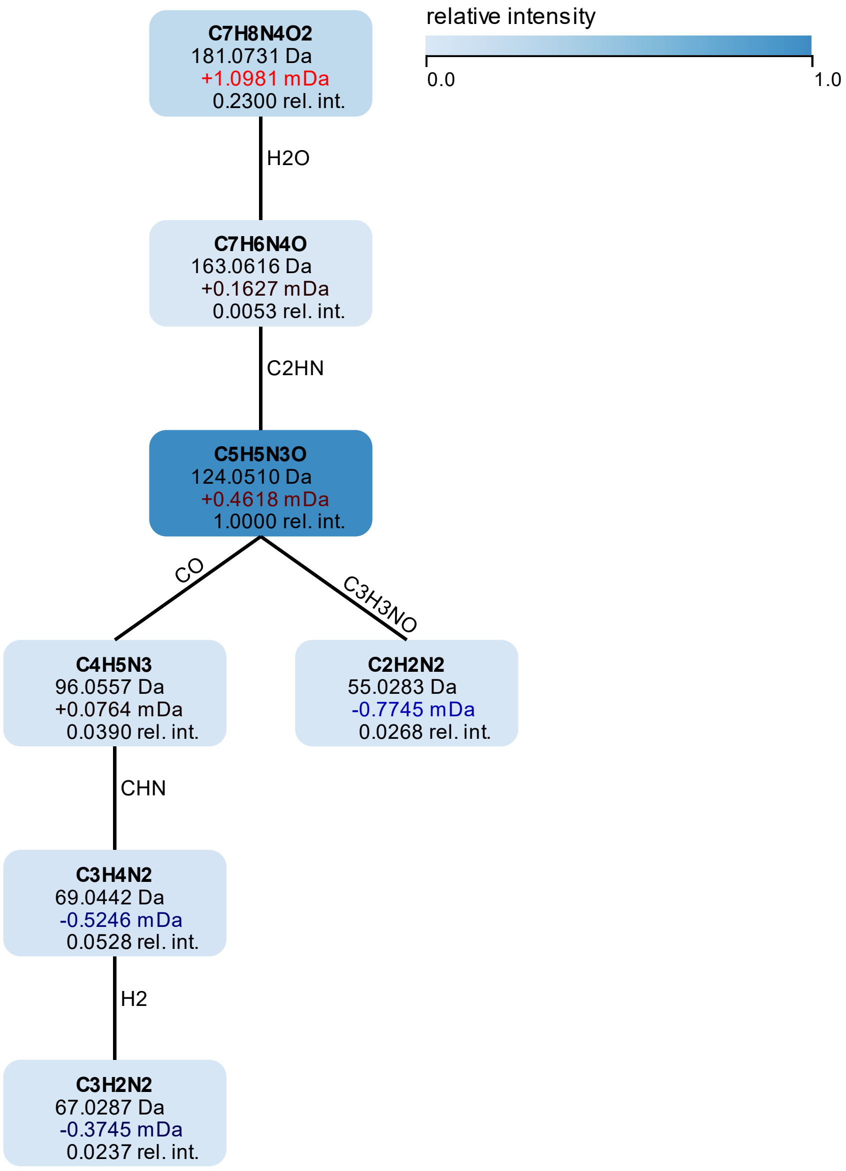

Table S16. First three predicted molecular formulas for paraxanthine low mobility species in water/acetonitrile (20/80).

Rank	Molecular formula	Adduct	Sirius score (%)	Tree score
1	$C_7H_8N_4O_2$	[M + H]+	98.715	16.21307
2	$C_9H_{10}NO_3$	[M + H]+	0.405	10.71741
3	$C_6H_{13}ClN_2O_2$	[M + H]+	0.317	10.47044

Table S17. First five predicted structures for molecular formula $C_7H_8N_4O_2$ (correct structure is marked with green) for paraxanthine low mobility species in water/acetonitrile (20/80).

Rank	Structure	CSI:FingerIDScore
1	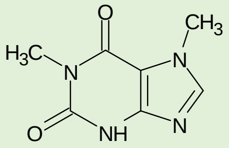	-28.25322
2	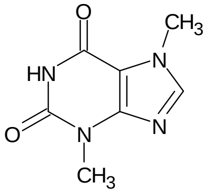	-38.52571
3	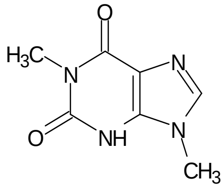	-47.60828
4	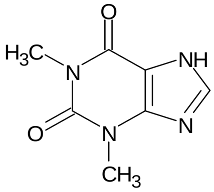	-59.03462
5	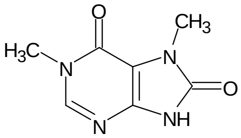	-65.54504

Figure S43. Fragmentation tree of molecular formula $C_7H_8N_4O_2$ for paraxanthine low mobility species in water/acetonitrile (20/80).

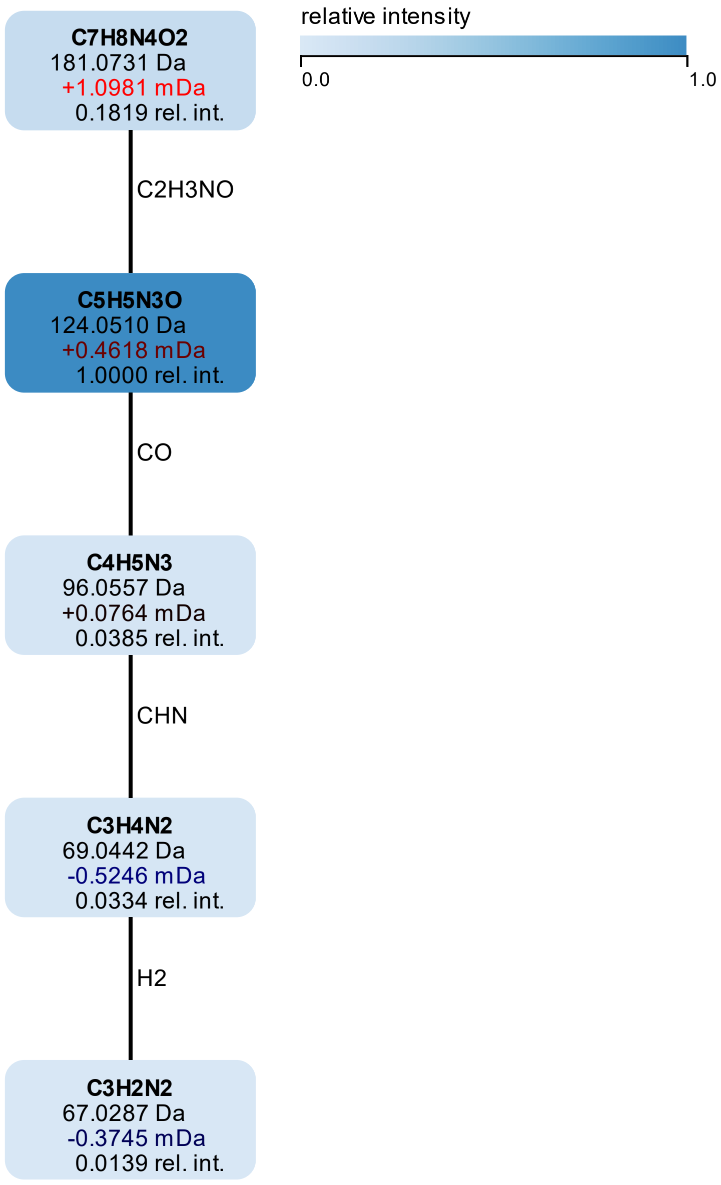

Table S18. First three predicted molecular formulas for theobromine high mobility species in water/acetonitrile (20/80).

Rank	Molecular formula	Adduct	Sirius score (%)	Tree score
1	$C_7H_8N_4O_2$	[M + H]+	100.00	70.96090
2	$C_5H_{13}N_2O_3P$	[M + H]+	0.00	49.28350
3	$C_9H_{10}NO_3$	[M + H]+	0.00	37.12909

Table S19. First five predicted structures for molecular formula $C_7H_8N_4O_2$ (correct structure is marked with green) for theobromine high mobility species in water/acetonitrile (20/80).

Rank	Structure	CSI:FingerIDScore
1	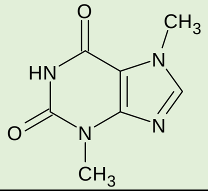	-8.13245
2	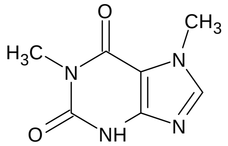	-33.93165
3	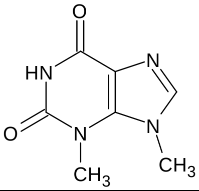	-35.68028
4	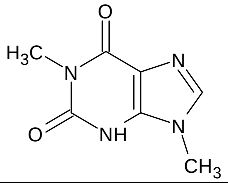	-51.33109
5	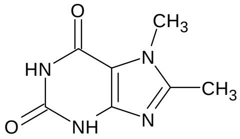	-62.34404

Figure S44. Fragmentation tree of molecular formula $C_7H_8N_4O_2$ for theobromine high mobility species in water/acetonitrile (20/80).

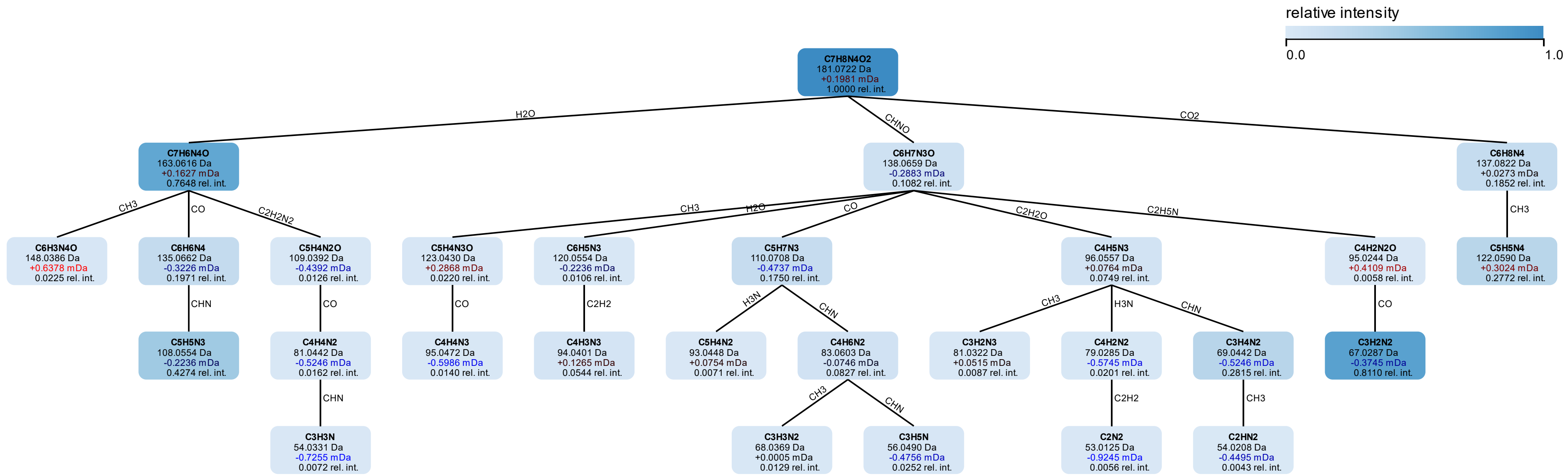

Table S20. First three predicted molecular formulas for theobromine low mobility species in water/acetonitrile (20/80).

Rank	Molecular formula	Adduct	Sirius score (%)	Tree score
1	$C_7H_8N_4O_2$	[M + H]+	100.00	46.53869
2	$C_5H_{13}N_2O_3P$	[M + H]+	0.00	33.91062
3	$C_7H_{10}BN_2OS$	[M + H]+	0.00	26.95168

Table S21. First five predicted structures for molecular formula $C_7H_8N_4O_2$ (correct structure is marked with green) for theobromine low mobility species in water/acetonitrile (20/80).

Rank	Structure	CSI:FingerIDScore
1	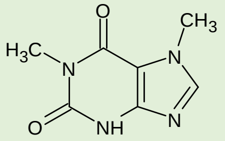	-23.75032
2	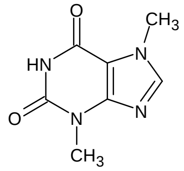	-42.58959
3	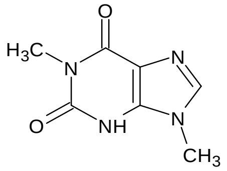	-49.53005
4	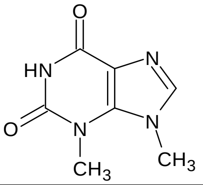	-58.34942
5	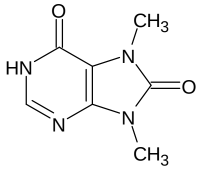	-66.71204

Figure S45. Fragmentation tree of molecular formula $C_7H_8N_4O_2$ for theobromine low mobility species in water/acetonitrile (20/80).

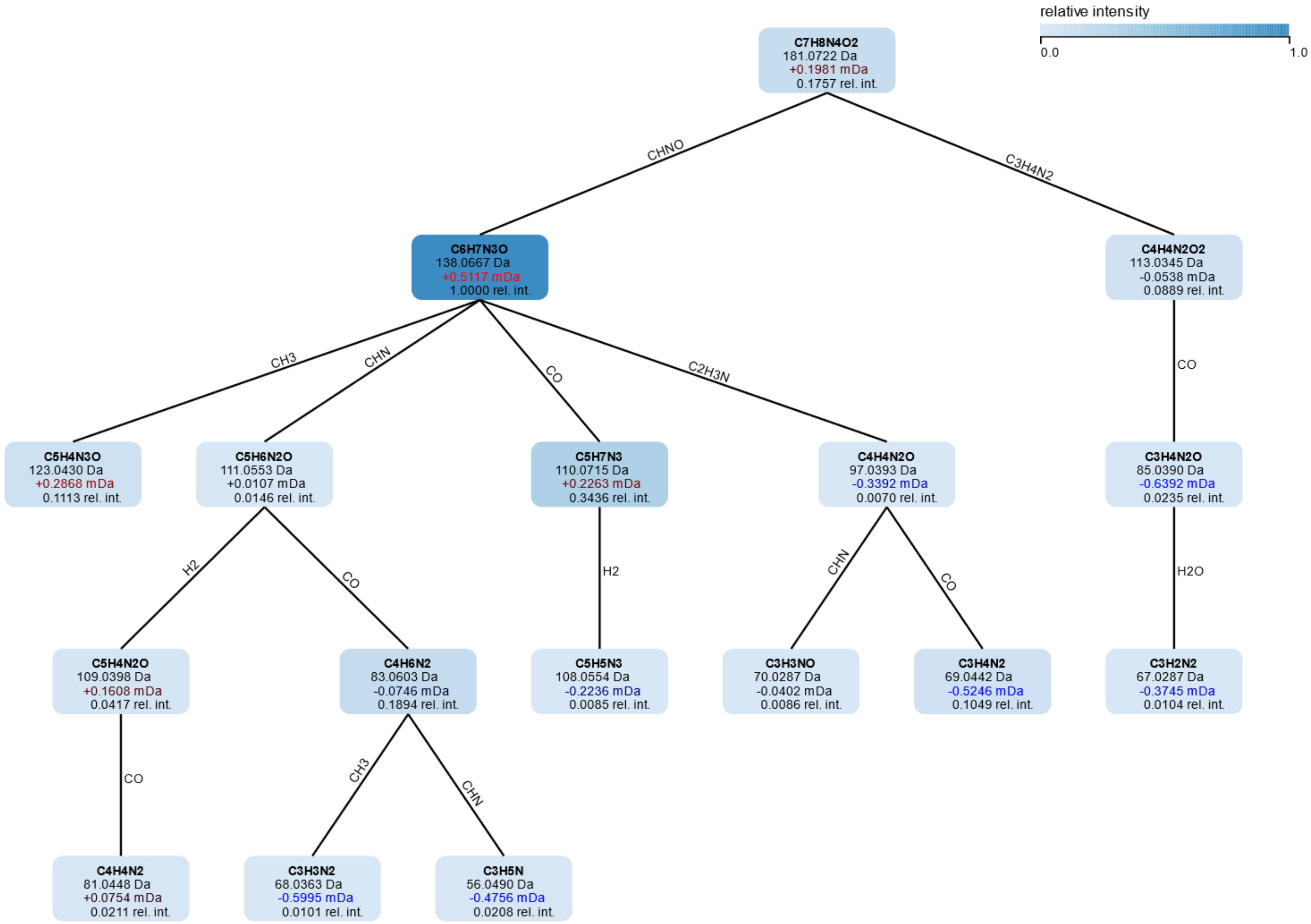

Table S22. First three predicted molecular formulas for paraxanthine (both peaks) in water/acetonitrile (20/80).

Rank	Molecular formula	Adduct	Sirius score (%)	Tree score
1	$C_7H_8N_4O_2$	[M + H]+	99.234	16.749881634799408
2	$C_9H_{10}NO_3$	[M + H]+	0.242	10.732220297712026
3	$C_6H_{13}ClN_2O_2$	[M + H]+	0.188	10.479002391236682

Table S23. First five predicted structures for molecular formula $C_7H_8N_4O_2$ (correct structure is marked with green) for paraxanthine (both peaks) in water/acetonitrile (20/80).

Rank	Structure	CSI:FingerIDScore
1	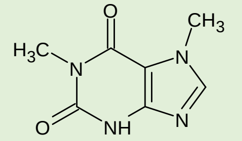	-25.70975
2	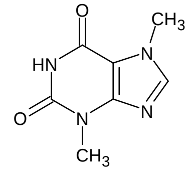	-35.97121
3	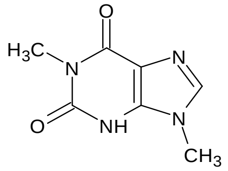	-44.62516
4	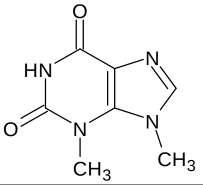	-65.05770
5	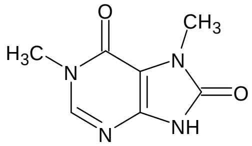	-65.36297

Figure S46. Fragmentation tree of molecular formula $C_7H_8N_4O_2$ for paraxanthine (both peaks) in water/acetonitrile (20/80).

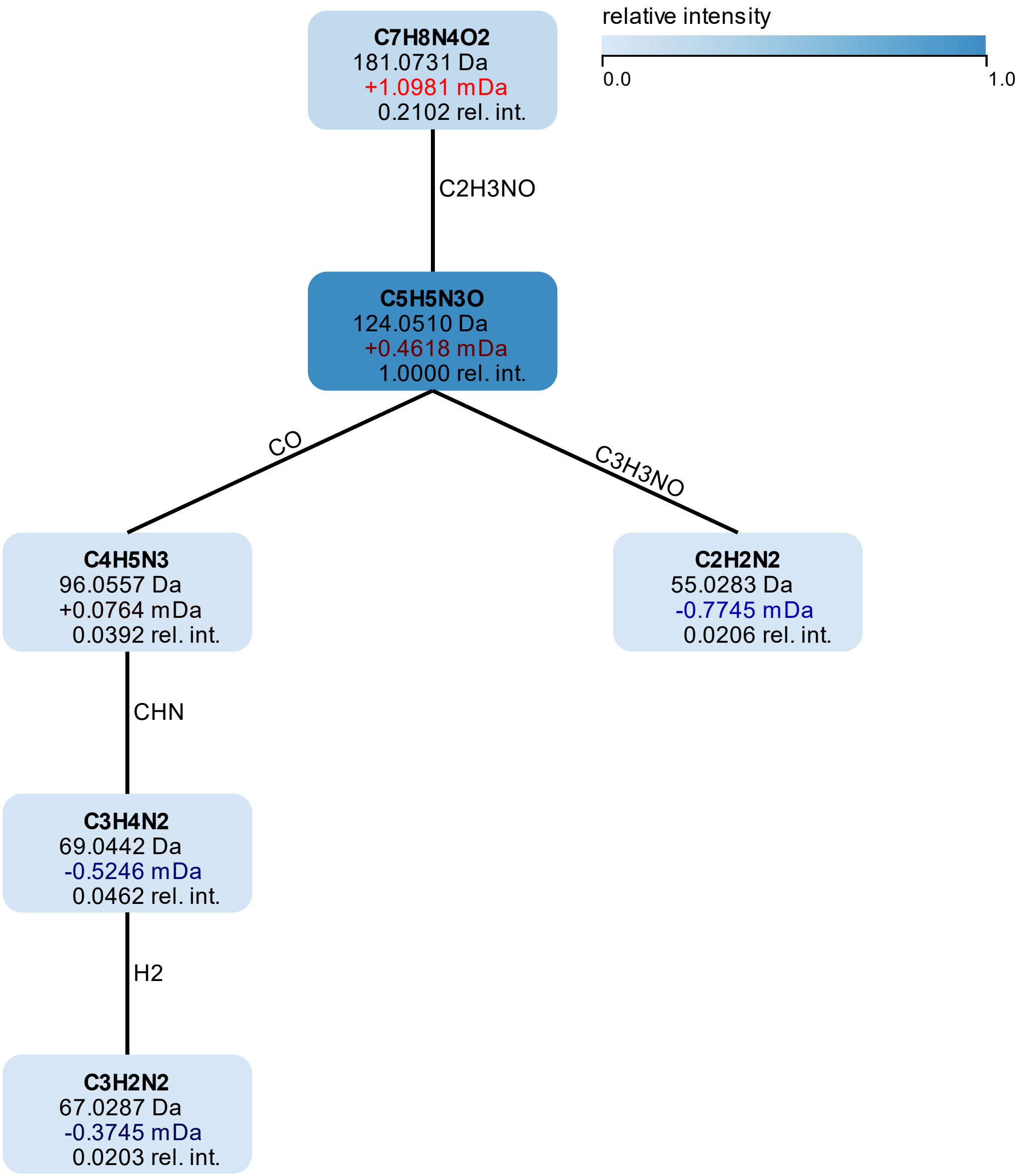

Table S24. First three predicted molecular formulas for paraxanthine (both peaks) in water.

Rank	Molecular formula	Adduct	Sirius score (%)	Tree score
1	$C_7H_8N_4O_2$	[M + H]+	99.953	37.44225
2	$C_6H_{13}ClN_2O_2$	[M + H]+	0.020	28.93197
3	$C_5H_{13}N_2O_3P$	[M + H]+	0.019	28.86293

Table S25. First five predicted structures for molecular formula $C_7H_8N_4O_2$ (correct structure is marked with green) for paraxanthine (both peaks) in water.

Rank	Structure	CSI:FingerIDScore
1	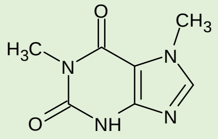	-9.037298
2	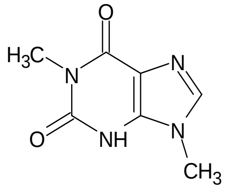	-27.764367
3	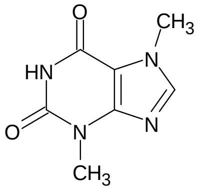	-32.180125
4	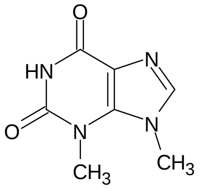	-50.356627
5	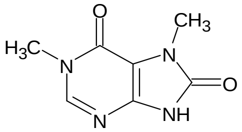	-51.973185

Figure S47. Fragmentation tree of molecular formula $C_7H_8N_4O_2$ for paraxanthine (both peaks) in water.

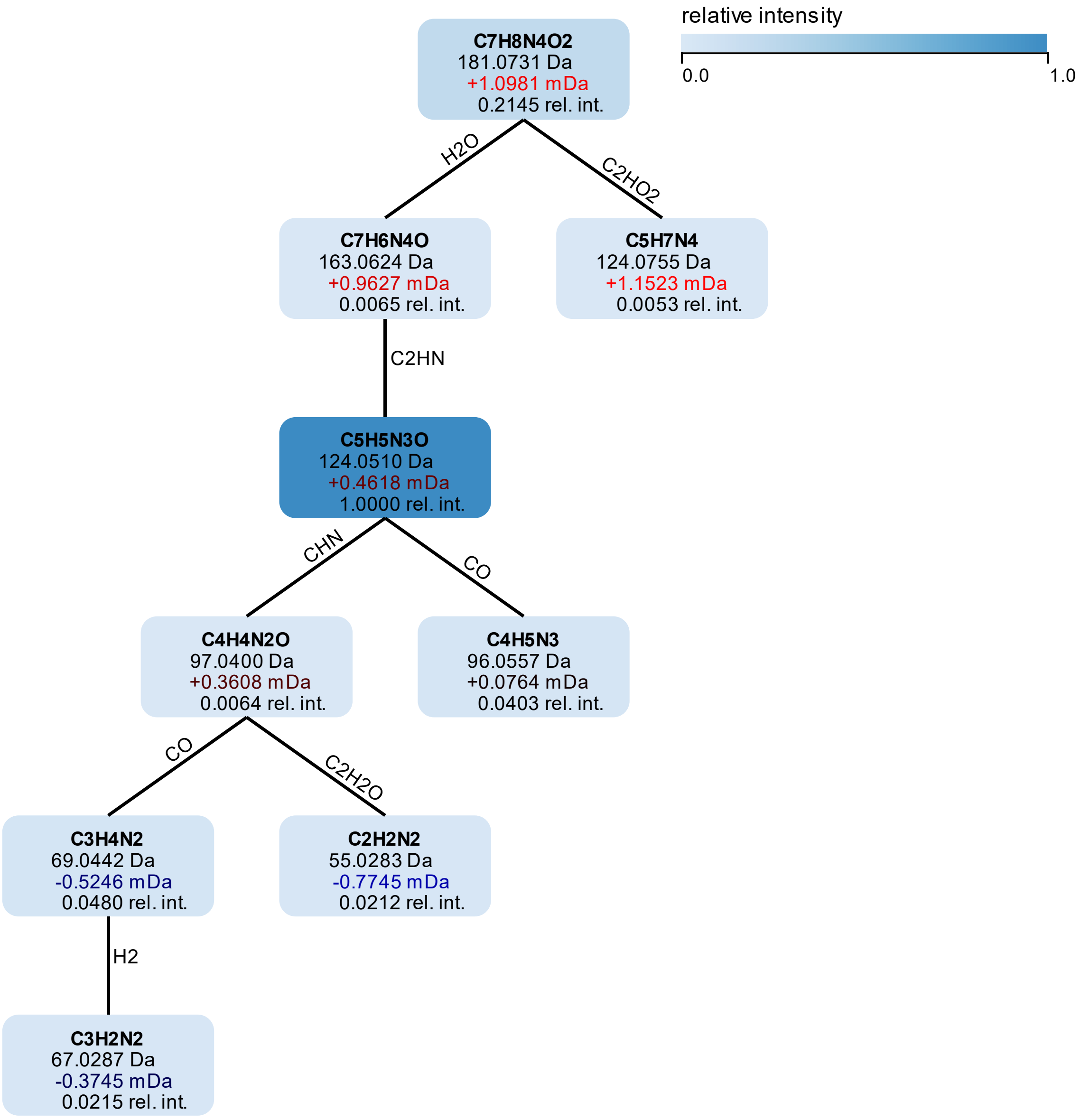

Table S26. First three predicted molecular formulas for paraxanthine (both peaks) in acetonitrile.

Rank	Molecular formula	Adduct	Sirius score (%)	Tree score
1	$C_7H_8N_4O_2$	[M + H]+	99.998	28.07309
2	$C_6H_{13}ClN_2O_2$	[M + H]+	0.005	18.07390
3	$C_7H_4B_2N_4O$	[M + H]+	0.004	17.87942

Table S27. First five predicted structures for molecular formula $C_7H_8N_4O_2$ (correct structure is marked with green) for paraxanthine (both peaks) in acetonitrile.

Rank	Structure	CSI:FingerIDScore
1	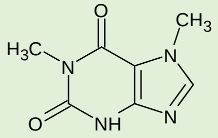	-18.80309
2	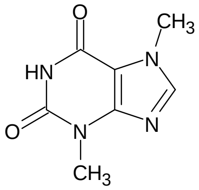	-33.62130
3	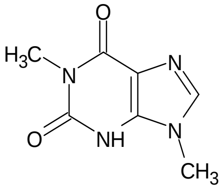	-37.19012
4	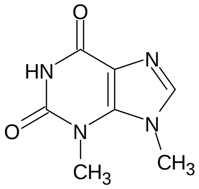	-56.30966
5	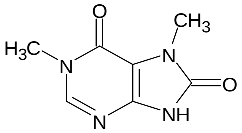	-61.78324

Figure S48. Fragmentation tree of molecular formula $C_7H_8N_4O_2$ for paraxanthine (both peaks) in acetonitrile.

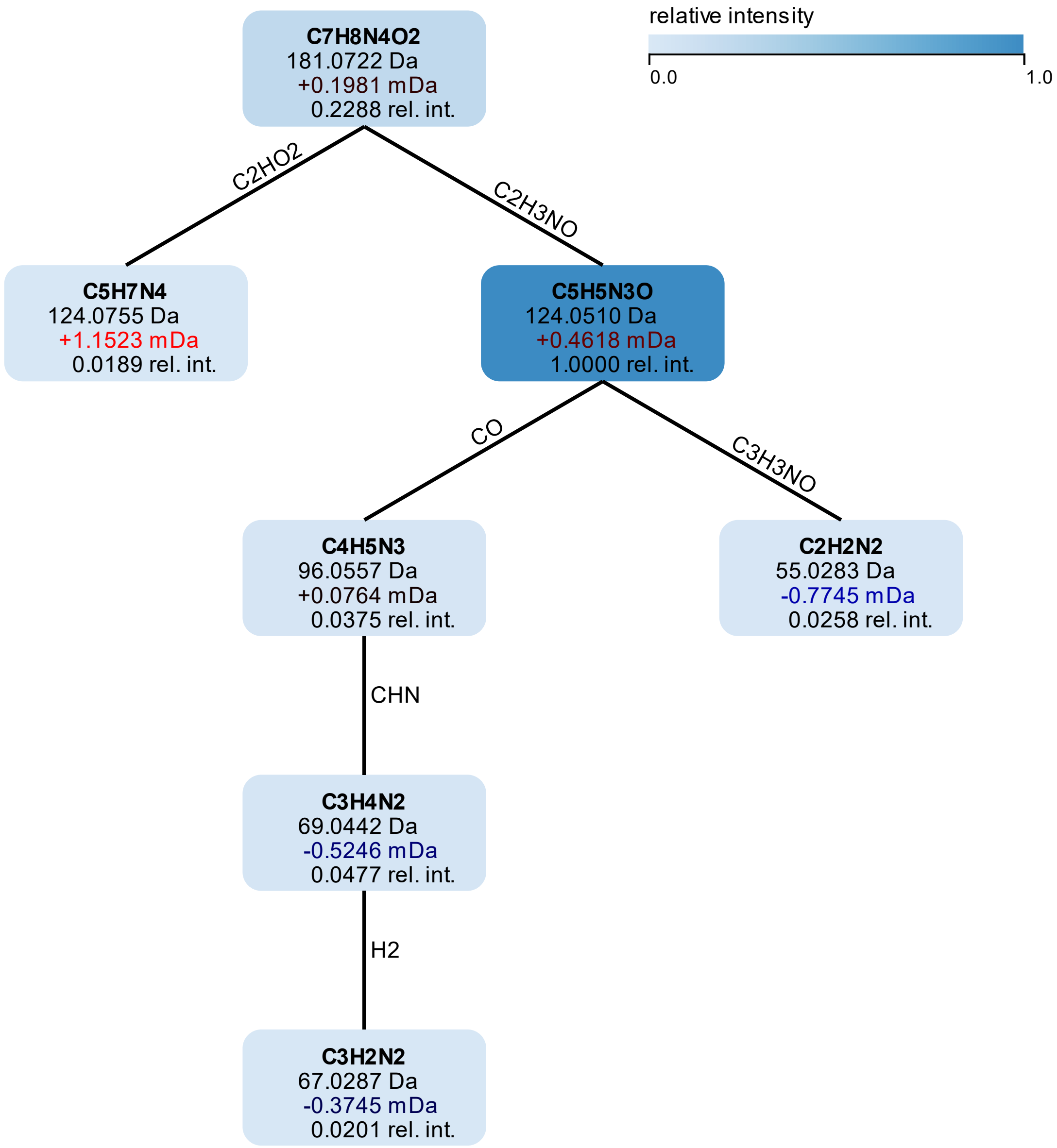

Table S28. First three predicted molecular formulas for theobromine (both peaks) in water/acetonitrile (20/80).

Rank	Molecular formula	Adduct	Sirius score (%)	Tree score
1	$C_7H_8N_4O_2$	[M + H]+	100.00	89.72466
2	$C_5H_{13}N_2O_3P$	[M + H]+	0.0	70.99099
3	$C_7H_{14}N_2O$	[M + K]+	0.0	57.99992

Table S29. First five predicted structures for molecular formula $C_7H_8N_4O_2$ (correct structure is marked with green) for theobromine (both peaks) in water/acetonitrile (20/80).

Rank	Structure	CSI:FingerIDScore
1	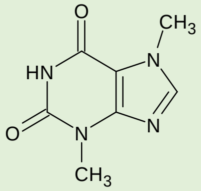	-7.208927
2	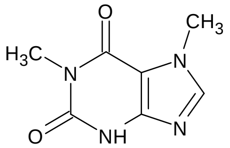	-33.061196
3	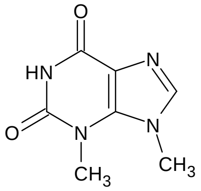	-34.908530
4	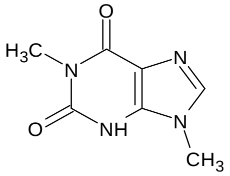	-50.617671
5	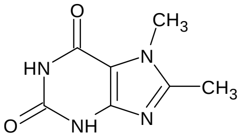	-65.892501

Figure S49. Fragmentation tree of molecular formula $C_7H_8N_4O_2$ for theobromine (both peaks) in water/acetonitrile (20/80).

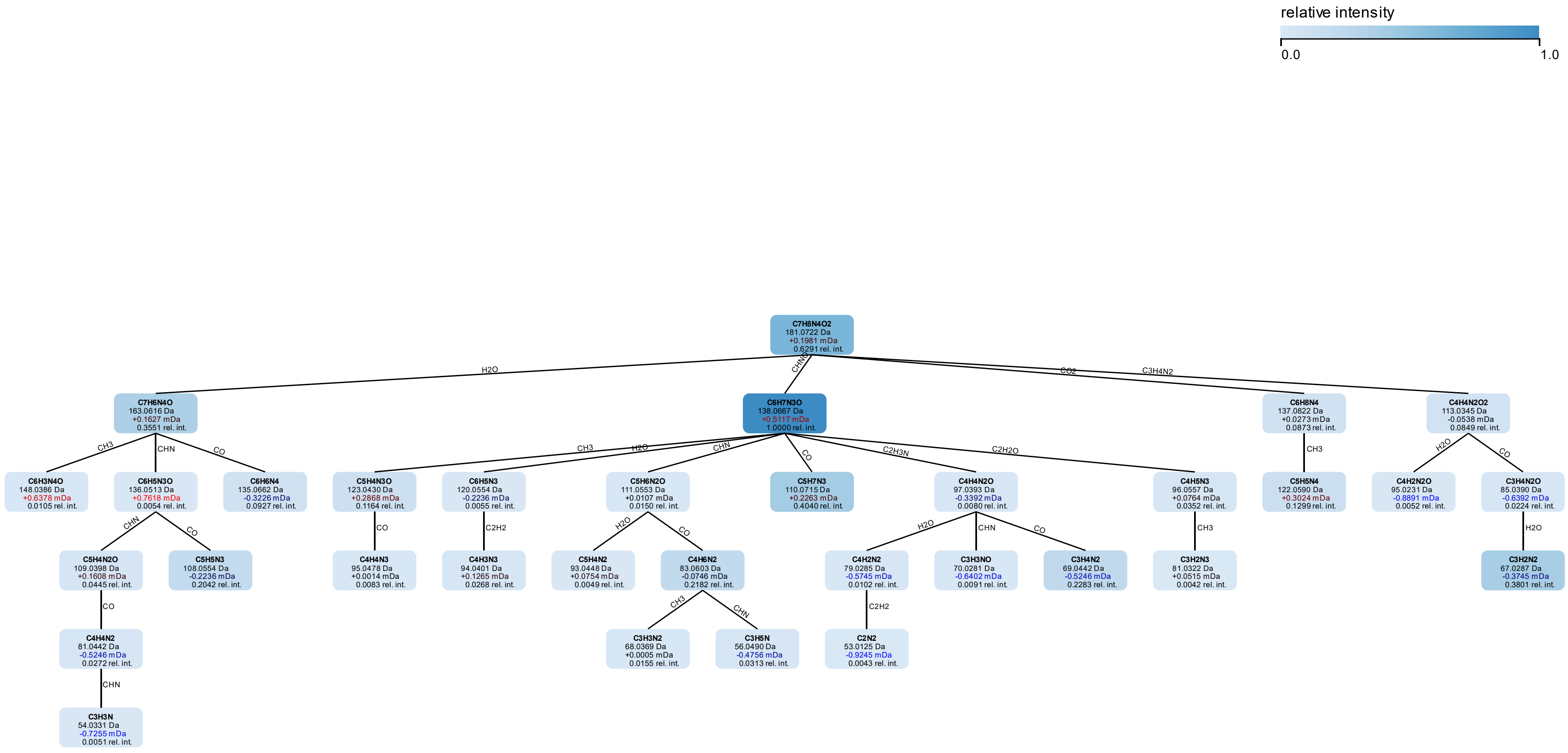

Table S30. First three predicted molecular formulas for theobromine (both peaks) in water.

Rank	Molecular formula	Adduct	Sirius score (%)	Tree score
1	$C_7H_8N_4O_2$	[M + H]+	100.00	68.65528
2	$C_5H_{13}N_2O_3P$	[M + H]+	0.0	48.67810
3	$C_6H_{13}ClN_2O_2$	[M + H]+	0.0	41.77558

Table S31. First five predicted structures for molecular formula $C_7H_8N_4O_2$ (correct structure is marked with green) for theobromine (both peaks) in water.

Rank	Structure	CSI:FingerIDScore
1	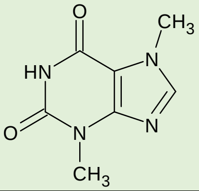	-7.920225
2	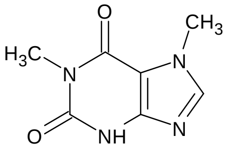	-33.833793
3	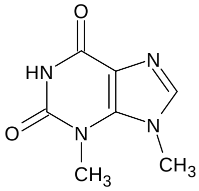	-35.825897
4	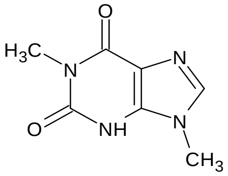	-51.595783
5	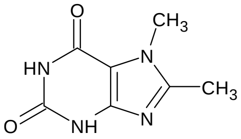	-62.552270

Figure S50. Fragmentation tree of molecular formula $C_7H_8N_4O_2$ for theobromine (both peaks) in water.

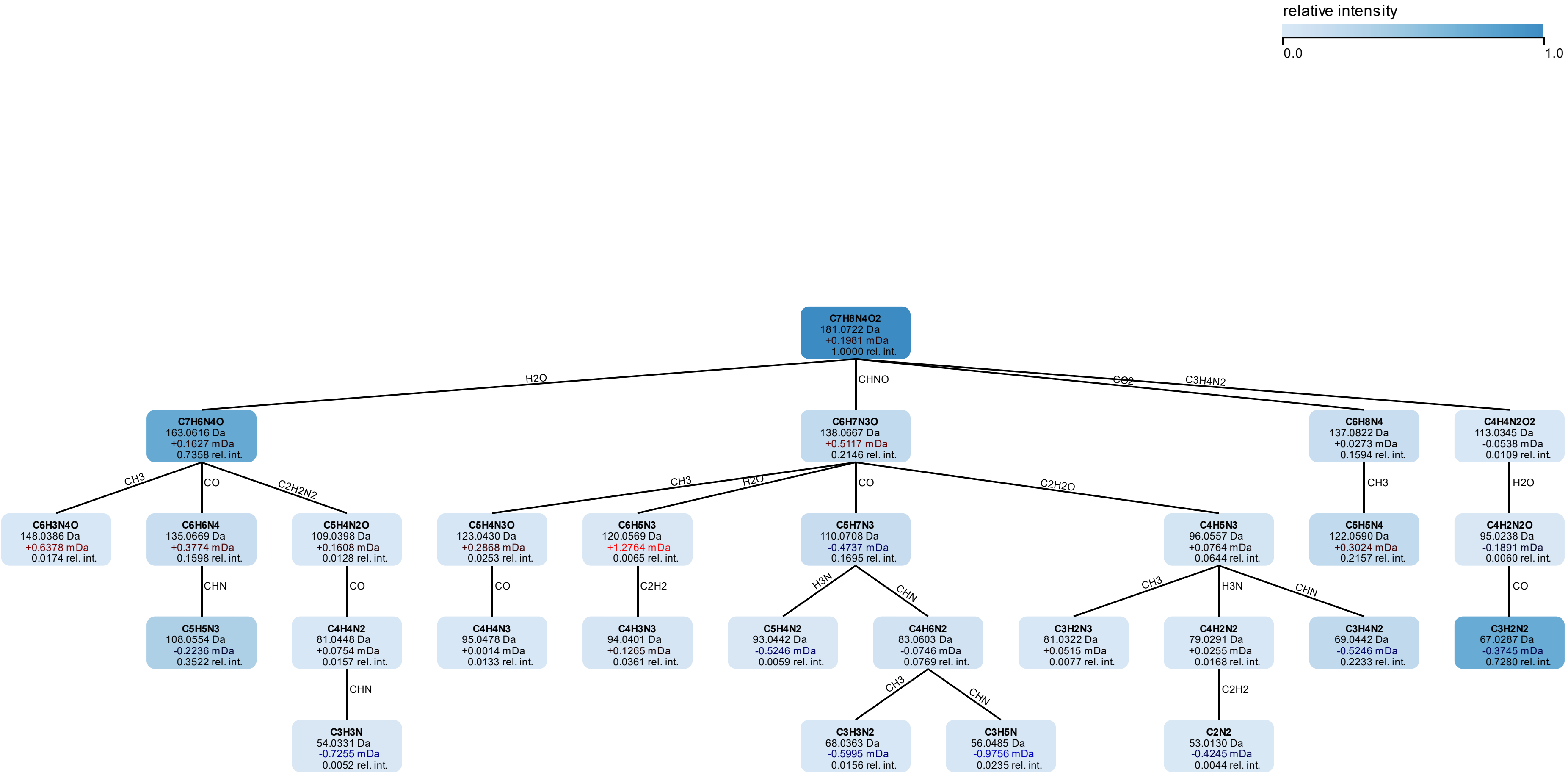

Table S32. First three predicted molecular formulas for theobromine (both peaks) in acetonitrile.

Rank	Molecular formula	Adduct	Sirius score (%)	Tree score
1	$C_7H_8N_4O_2$	[M + H]+	100.00	40.37399
2	$C_5H_{13}N_2O_3P$	[M + H]+	0.0	26.46304
3	$C_6H_{13}ClN_2O_2$	[M + H]+	0.0	25.51799

Table S33. First five predicted structures for molecular formula $C_7H_8N_4O_2$ (correct structure is marked with green) for theobromine (both peaks) in acetonitrile.

Rank	Structure	CSI:FingerIDScore
1	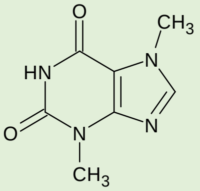	-13.04622
2	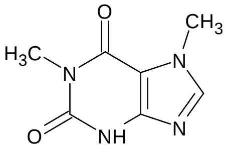	-34.82588
3	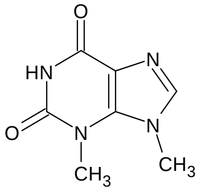	-39.27857
4	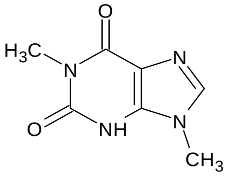	-50.95333
5	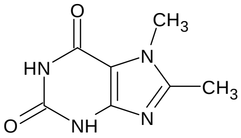	-68.39981

Figure S51. Fragmentation tree of molecular formula $C_7H_8N_4O_2$ for theobromine (both peaks) in acetonitrile.

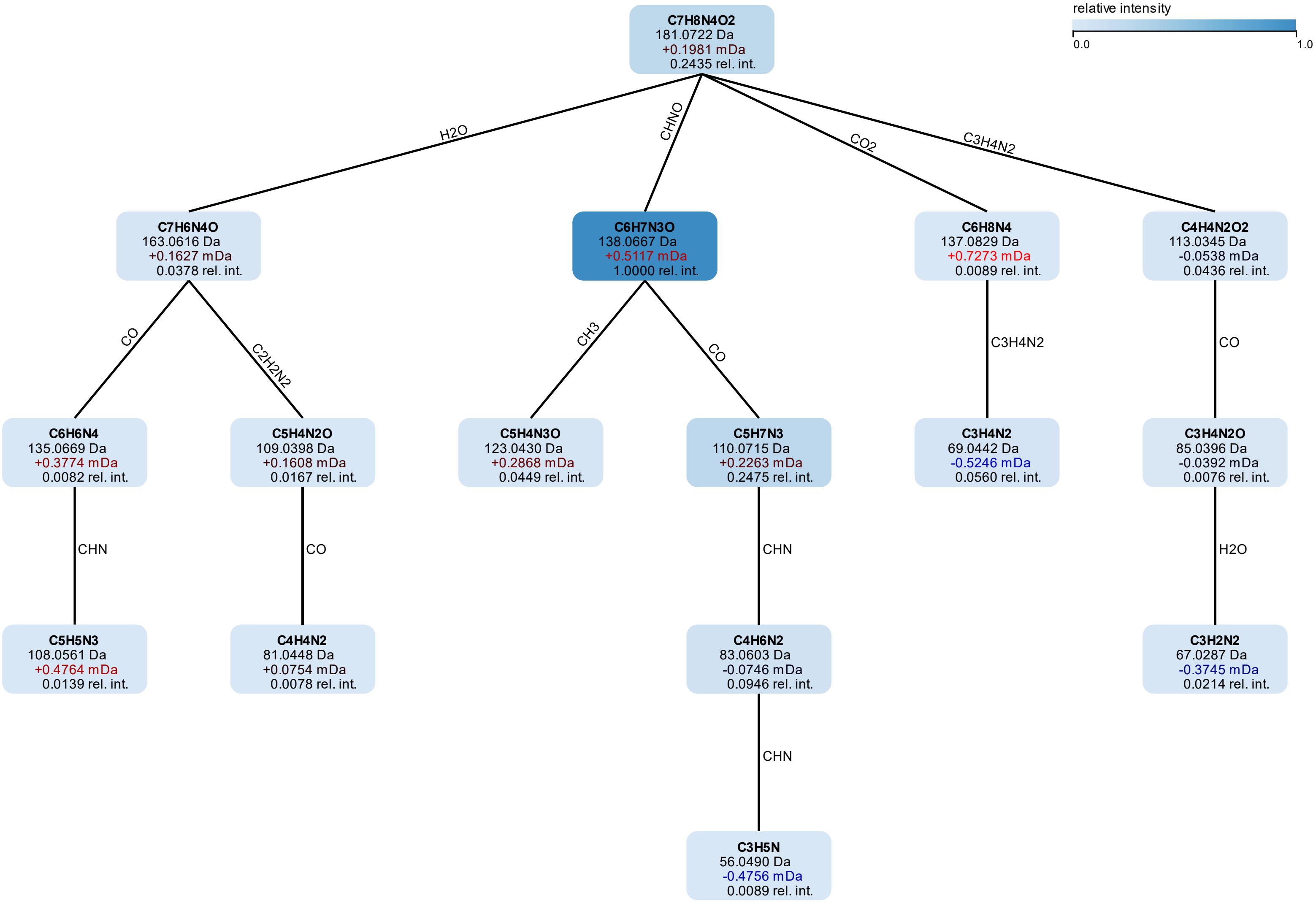

Table S34. First three predicted molecular formulas for theophylline in water/acetonitrile (20/80).

Rank	Molecular formula	Adduct	Sirius score (%)	Tree score
1	$C_7H_8N_4O_2$	[M + H]+	99.453	30.22079
2	$C_9H_{10}NO_3$	[M + H]+	0.455	24.83369
3	$C_6H_{13}ClN_2O_2$	[M + H]+	0.055	22.71237

Table S35. First five predicted structures for molecular formula $C_7H_8N_4O_2$ (correct structure is marked with green) for theophylline in water/acetonitrile (20/80).

Rank	Structure	CSI:FingerIDScore
1	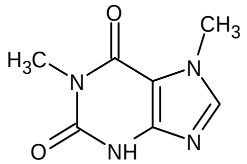	-42.57445
2	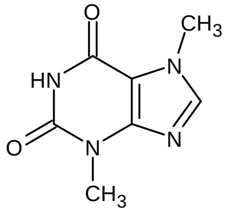	-46.16612
3	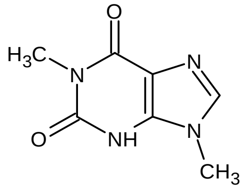	-58.47485
4	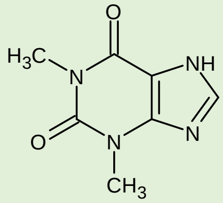	-65.43552
5	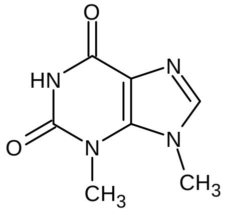	-71.96505

Figure S52. Fragmentation tree of molecular formula $C_7H_8N_4O_2$ for theophylline in water/acetonitrile (20/80).

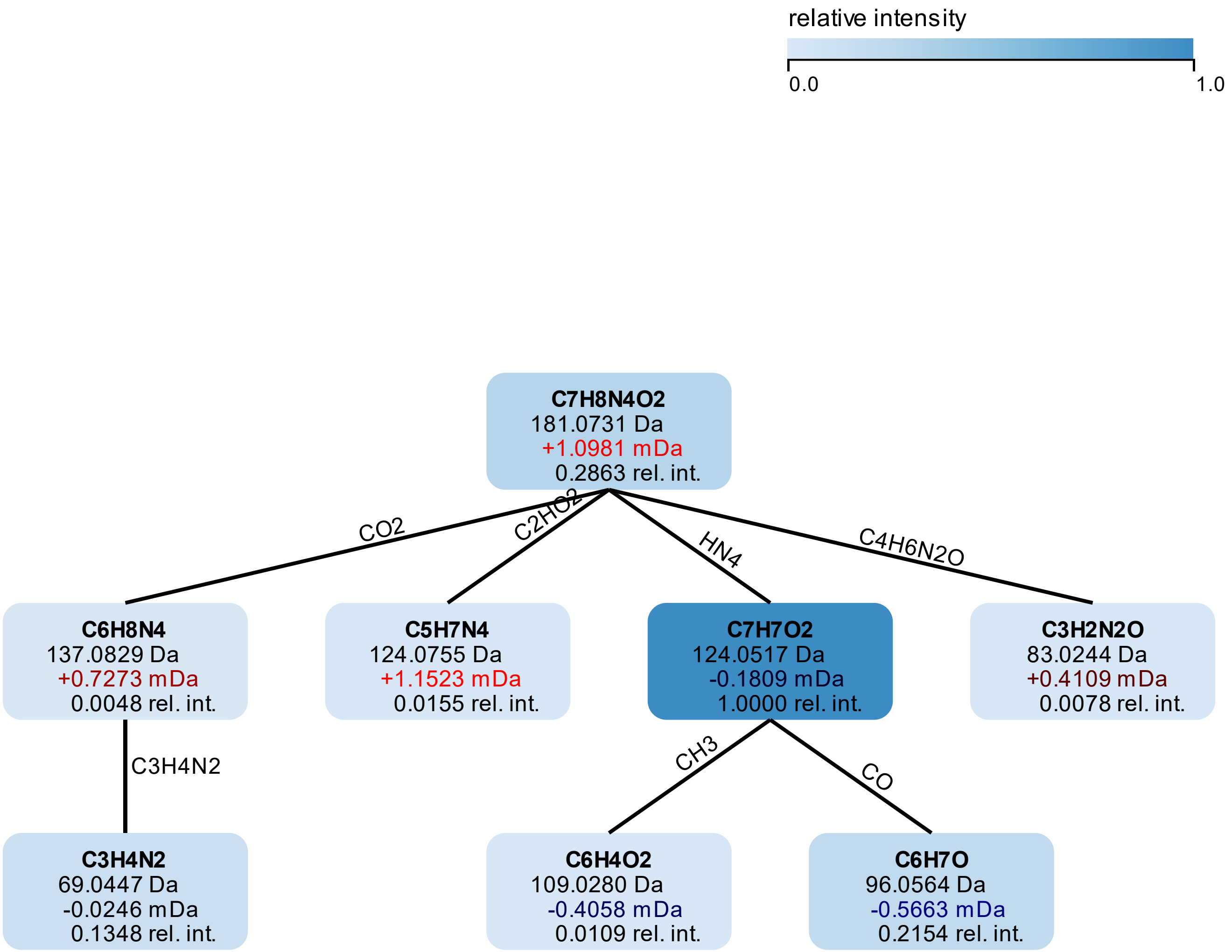

Ionization efficiency experiments and results

The abundance of a metabolite in a sample is predominantly assessed based on a peak area. The intensity of the MS peak of the metabolites is greatly affected by their ionization efficiency in the electrospray. We and other groups have previously evaluated the effect of the compound structure on its ionization efficiency;[21,22] however, the effect of protomer formation on the ionization efficiency has not been investigated.

The ionization efficiency values for caffeine and its metabolites in 5 different mobile phases were measured according to the previously published method by Liigand et al.[23] Ionization efficiency measurements were carried out under following parameters: solutions with concentrations in the range of 6.6 – 61.2 nmol/mL were prepared in 6 different solvent compositions - neat MeCN, MeCN/H_2O 80/20, MeCN/H_2O 60/40, MeCN/H_2O 40/60, MeCN/H_2O 20/80 and neat H_2O. Flow injection measurements were conducted with isocratic eluent with all six solvent compositions mentioned above with flow rate of 0.3 mL/min. Mass spectra were acquired in V-mode for one minute over a range of *m/z* 50.0000 to 1200.0000 Da under the following experiment parameters: source temperature 100 °C, desolvation temperature 400 °C, the capillary voltage was optimized in the range of 1.5 to 2.6 kV, desolvation gas flow rate 600 L/h, nebulizer gas pressure 6.0 bar. Injection volume was altered to obtain calibration graph – volumes of 2,4,6, 8, and 10 µL were injected.

Calibration graphs were obtained from acquired data to determine ionization efficiencies. Isotope distribution was calculated based on SMILES and considered for signal corrections. Linearity was checked for all compounds – a measured datapoint was removed if the residual was larger than 0.05; however, minimum three datapoints had to meet the requirements to calculate slope. All IE values were anchored with tetraethylammonium chloride measured in different solvents (log*IE* = 3.95 in all solvents). Three rounds of measurements were performed for caffeine metabolites over time period of two months. For guanine and adenine, two rounds of measurements and uric acid derivatives one round of measurements were performed. For some compounds, measurements in acetonitrile could not be conducted due to the low solubility of the compound.

Two compounds, theobromine and 3-methylxanthine, had previously shown an alteration of the proportion of protomers formed with acetonitrile content in the mobile phase. Therefore, if protomer formation affected ionization efficiency we would expect to see a significantly different ionization efficiency pattern for theobromine and 3-methylxanthine in comparison to other compounds for which either only one protomer was observed or the ratio did not depend on the acetonitrile content.

The change in measured ionization efficiency values with mobile phase composition showed a very similar profile for all compounds, independent of the protomer formation, see Figures S6.1.-6.3. Therefore, it can be concluded that ionization efficiency is insignificantly affected by the protomer formation. This could indicate that (1) ionization efficiency and protomer formation

are limited by different steps in the ionization process or (2) that the physiochemical properties of the protomers are sufficiently similar from ionization efficiency point of view to yield indistinguishable ionization efficiency values.

The measured ionization efficiency values ranged from 1.60 to 5.97 and the largest variation in log*IE* value was observed for hypoxanthine, from 4.48 (100% MeCN) to 5.94 (0% MeCN). The largest log*IE* values were also observed for hypoxanthine, which is the strongest base in the dataset. Regarding solvent effect an increase in the ionization efficiency values in 100% of acetonitrile was observed; however, the measurements in pure acetonitrile were less stable and quantitative results could be obtained only for some of the studied compounds. Generally, solvents with higher organic modifier content are known to yield higher ionization efficiency values due to increased evaporation of the organic solvent and which facilitates formation of the smaller droplets in ESI.[23,24]

Figure S53. Ionization efficiencies for caffeine metabolites in positive mode anchored with tetraethylammonium chloride.

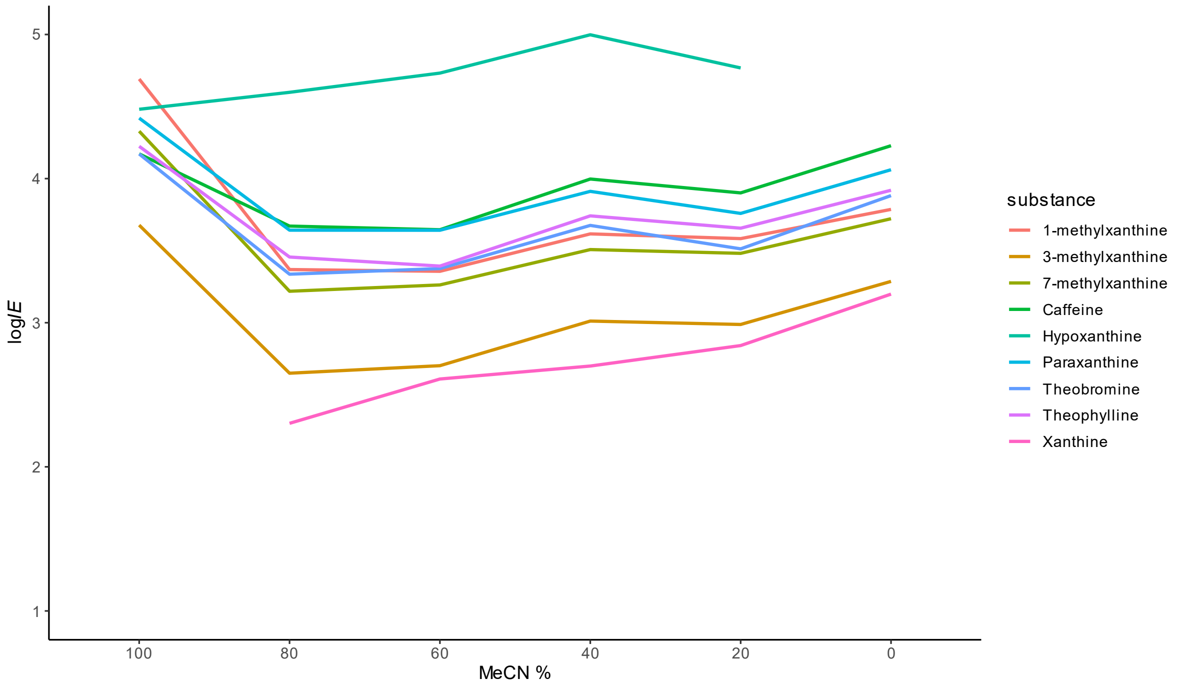

Figure S54. Ionization efficiencies for uric acid metabolites in positive mode anchored with tetraethylammonium chloride.

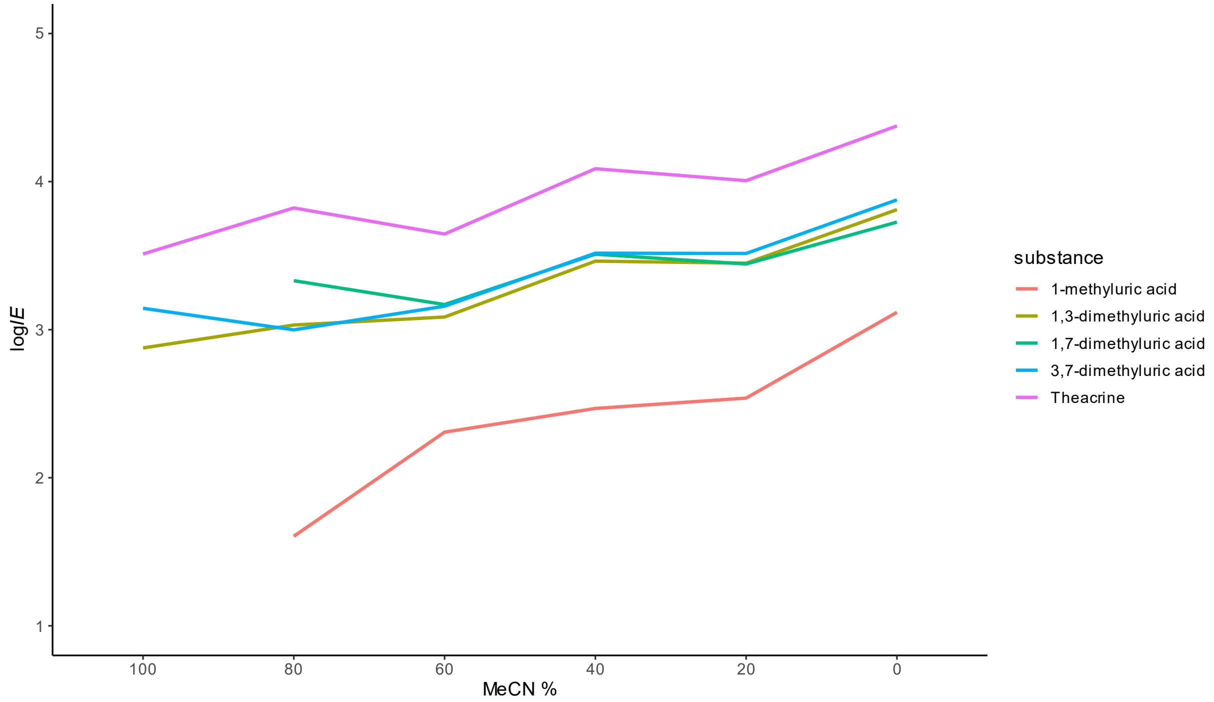

Figure S55. Ionization efficiencies for nucleic bases in positive mode anchored with tetraethylammonium chloride.

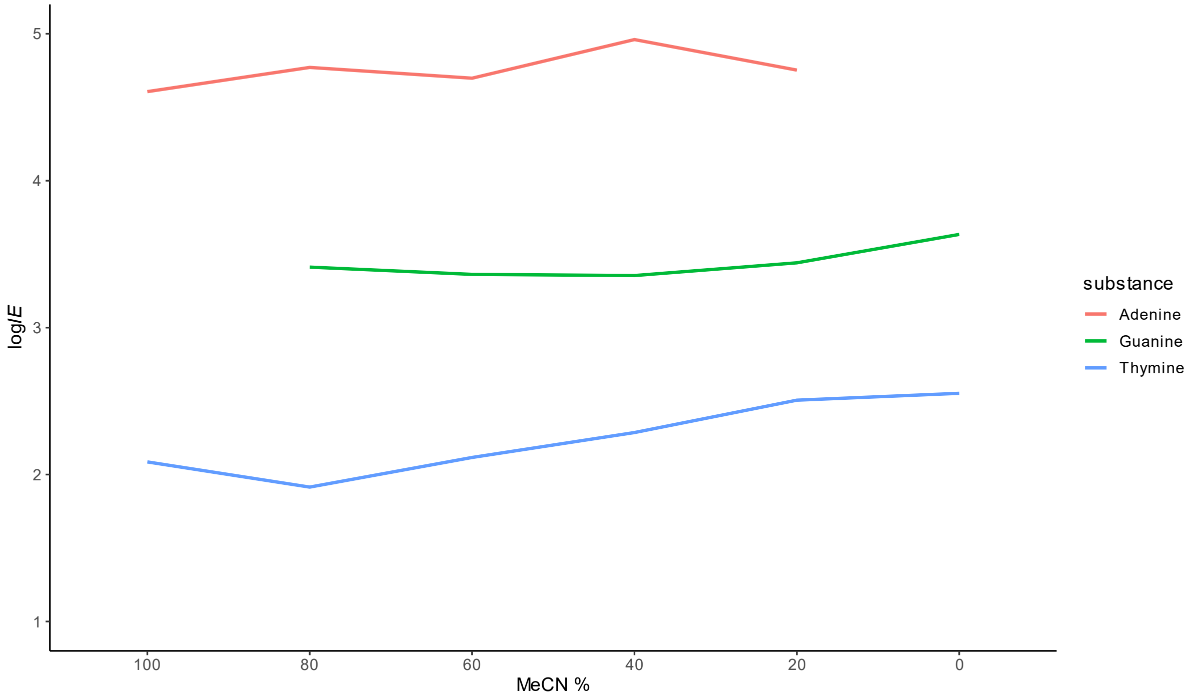

Table S36. Mean values of measured ionization efficiencies in six different solvent compositions.

Compound	Solvent	log*IE*	STDEV
Caffeine	H2O	2.20E+07	7.48E+06
Caffeine	H2O:MeCN 20:80	1.27E+07	4.83E+06
Caffeine	H2O:MeCN 40:60	1.19E+07	6.28E+06
Caffeine	H2O:MeCN 60:40	1.59E+07	6.94E+06
Caffeine	H2O:MeCN 80:20	1.90E+07	7.38E+06
Caffeine	MeCN	1.20E+08	1.70E+06
Theophylline	H2O	9.74E+06	2.16E+06
Theophylline	H2O:MeCN 20:80	6.67E+06	2.26E+06
Theophylline	H2O:MeCN 40:60	5.98E+06	1.53E+06
Theophylline	H2O:MeCN 60:40	7.48E+06	1.51E+06
Theophylline	H2O:MeCN 80:20	8.76E+06	1.08E+06
Theophylline	MeCN	1.12E+08	3.14E+07
Paraxanthine	H2O	1.27E+07	1.89E+06
Paraxanthine	H2O:MeCN 20:80	9.57E+06	4.59E+06
Paraxanthine	H2O:MeCN 40:60	8.80E+06	1.37E+06
Paraxanthine	H2O:MeCN 60:40	1.09E+07	1.86E+06
Paraxanthine	H2O:MeCN 80:20	1.22E+07	2.50E+06
Paraxanthine	MeCN	2.04E+08	9.28E+06
Theobromine	H2O	1.02E+07	3.80E+06
Theobromine	H2O:MeCN 20:80	4.58E+06	1.94E+06
Theobromine	H2O:MeCN 40:60	4.78E+06	6.39E+05
Theobromine	H2O:MeCN 60:40	6.14E+06	9.57E+05
Theobromine	H2O:MeCN 80:20	8.23E+06	3.40E+06
Theobromine	MeCN	1.04E+08	1.99E+07
1-methylxanthine	H2O	6.83E+06	1.10E+06
1-methylxanthine	H2O:MeCN 20:80	7.40E+06	4.33E+06
1-methylxanthine	H2O:MeCN 40:60	5.33E+06	1.47E+06
1-methylxanthine	H2O:MeCN 60:40	6.57E+06	2.41E+06
1-methylxanthine	H2O:MeCN 80:20	7.75E+06	1.78E+06
3-methylxanthine	H2O	2.50E+06	8.32E+05
3-methylxanthine	H2O:MeCN 20:80	1.76E+06	1.72E+06
3-methylxanthine	H2O:MeCN 40:60	1.63E+06	9.34E+05
3-methylxanthine	H2O:MeCN 60:40	2.36E+06	1.74E+06
3-methylxanthine	H2O:MeCN 80:20	2.58E+06	1.32E+06
7-methylxanthine	H2O	6.25E+06	1.47E+06
7-methylxanthine	H2O:MeCN 20:80	4.36E+06	1.97E+06
7-methylxanthine	H2O:MeCN 40:60	3.53E+06	4.84E+05
7-methylxanthine	H2O:MeCN 60:40	5.06E+06	1.74E+06

7-methylxanthine	H2O:MeCN 80:20	5.84E+06	8.69E+05
Xanthine	H2O	1.68E+06	1.58E+05
Xanthine	H2O:MeCN 20:80	9.18E+05	8.83E+05
Xanthine	H2O:MeCN 40:60	1.36E+06	1.07E+06
Xanthine	H2O:MeCN 60:40	8.44E+05	3.26E+05
Xanthine	H2O:MeCN 80:20	1.16E+06	4.54E+04
Hypoxanthine	H2O	4.35E+08	6.12E+08
Hypoxanthine	H2O:MeCN 20:80	9.62E+07	2.00E+07
Hypoxanthine	H2O:MeCN 40:60	9.96E+07	2.19E+07
Hypoxanthine	H2O:MeCN 60:40	1.09E+08	2.29E+06
Hypoxanthine	H2O:MeCN 80:20	9.65E+07	1.08E+06
Guanine	H2O	4.99E+06	1.02E+06
Guanine	H2O:MeCN 20:80	4.66E+06	NA*
Guanine	H2O:MeCN 40:60	4.48E+06	6.72E+05
Guanine	H2O:MeCN 60:40	3.80E+06	1.94E+06
Guanine	H2O:MeCN 80:20	5.14E+06	7.87E+05
Adenine	H2O	4.65E+08	6.42E+08
Adenine	H2O:MeCN 20:80	7.97E+07	1.36E+07
Adenine	H2O:MeCN 40:60	8.02E+07	9.20E+06
Adenine	H2O:MeCN 60:40	9.51E+07	4.02E+06
Adenine	H2O:MeCN 80:20	9.12E+07	3.83E+06
Thymine	H2O	3.54E+05	NA*
Thymine	H2O:MeCN 20:80	1.24E+05	NA*
Thymine	H2O:MeCN 40:60	2.27E+05	NA*
Thymine	H2O:MeCN 60:40	2.07E+05	NA*
Thymine	H2O:MeCN 80:20	5.33E+05	NA*
Thymine	MeCN	9.74E+05	NA*
1-methyluric acid	H2O	1.30E+06	NA*
1-methyluric acid	H2O:MeCN 20:80	6.09E+04	NA*
1-methyluric acid	H2O:MeCN 40:60	3.53E+05	NA*
1-methyluric acid	H2O:MeCN 60:40	3.15E+05	NA*
1-methyluric acid	H2O:MeCN 80:20	5.73E+05	NA*
1,3-dimethyluric acid	H2O	6.40E+06	NA*
1,3-dimethyluric acid	H2O:MeCN 20:80	1.63E+06	NA*
1,3-dimethyluric acid	H2O:MeCN 40:60	2.12E+06	NA*
1,3-dimethyluric acid	H2O:MeCN 60:40	3.11E+06	NA*
1,3-dimethyluric acid	H2O:MeCN 80:20	4.67E+06	NA*
1,7-dimethyluric acid	H2O	5.27E+06	NA*
1,7-dimethyluric acid	H2O:MeCN 20:80	3.24E+06	NA*
1,7-dimethyluric acid	H2O:MeCN 40:60	2.57E+06	NA*
1,7-dimethyluric acid	H2O:MeCN 60:40	3.47E+06	NA*
1,7-dimethyluric acid	H2O:MeCN 80:20	4.61E+06	NA*

3,7-dimethyluric acid	H2O	7.45E+06	NA*
3,7-dimethyluric acid	H2O:MeCN 20:80	1.51E+06	NA*
3,7-dimethyluric acid	H2O:MeCN 40:60	2.51E+06	NA*
3,7-dimethyluric acid	H2O:MeCN 60:40	3.52E+06	NA*
3,7-dimethyluric acid	H2O:MeCN 80:20	5.43E+06	NA*
Theacrine	H2O	2.35E+07	NA*
Theacrine	H2O:MeCN 20:80	1.01E+07	NA*
Theacrine	H2O:MeCN 40:60	7.69E+06	NA*
Theacrine	H2O:MeCN 60:40	1.31E+07	NA*
Theacrine	H2O:MeCN 80:20	1.69E+07	NA*
Theacrine	MeCN	2.59E+07	NA*

* One measurement was performed; therefore, standard deviation could not be found.

References

(1) Klamt, A. Conductor-like Screening Model for Real Solvents: A New Approach to the Quantitative Calculation of Solvation Phenomena. *J. Phys. Chem.* **1995**, *99* (7), 2224–2235. https://doi.org/10.1021/j100007a062.

(2) Klamt, A.; Jonas, V.; Bürger, T.; Lohrenz, J. C. W. Refinement and Parametrization of COSMO-RS. *J. Phys. Chem. A* **1998**, *102* (26), 5074–5085. https://doi.org/10.1021/jp980017s.

(3) Eckert, F.; Klamt, A. Fast Solvent Screening via Quantum Chemistry: COSMO-RS Approach. *AIChE J.* **2002**, *48* (2), 369–385. https://doi.org/10.1002/aic.690480220.

(4) Klamt, A. The COSMO and COSMO-RS Solvation Models. *WIREs Comput. Mol. Sci.* **2018**, *8* (1). https://doi.org/10.1002/wcms.1338.

(5) Klamt, A.; Eckert, F.; Arlt, W. COSMO-RS: An Alternative to Simulation for Calculating Thermodynamic Properties of Liquid Mixtures. *Annu. Rev. Chem. Biomol. Eng.* **2010**, *1* (1), 101–122. https://doi.org/10.1146/annurev-chembioeng-073009-100903.

(6) Klamt, A.; Reinisch, J.; Eckert, F.; Hellweg, A.; Diedenhofen, M. Polarization Charge Densities Provide a Predictive Quantification of Hydrogen Bond Energies. *Phys Chem Chem Phys* **2012**, *14* (2), 955–963. https://doi.org/10.1039/C1CP22640A.

(7) Klamt, A.; Reinisch, J.; Eckert, F.; Graton, J.; Le Questel, J.-Y. Interpretation of Experimental Hydrogen-Bond Enthalpies and Entropies from COSMO Polarisation Charge Densities. *Phys. Chem. Chem. Phys.* **2013**, *15* (19), 7147. https://doi.org/10.1039/c3cp44611e.

(8) Gogia, S.; Jain, A.; Puranik, M. Structures, Ionization Equilibria, and Tautomerism of 6-Oxopurines in Solution. *J. Phys. Chem. B* **2009**, *113* (45), 15101–15118. https://doi.org/10.1021/jp9057753.

(9) Camiruaga, A.; Usabiaga, I.; D'mello, V. C.; García, G. A.; Wategaonkar, S.; Fernández, J. A. Revisiting the Spectroscopy of Xanthine Derivatives: Theobromine and Theophylline. *Phys. Chem. Chem. Phys.* **2019**, *21* (48), 26430–26437. https://doi.org/10.1039/C9CP05068J.

(10) Kim, D.; Yang, K. Y.; Kim, H. M.; Kim, T.-R.; Kim, N. J.; Shin, S.; Kim, S. K. Site-Dependent Effects of Methylation on the Electronic Spectra of Jet-Cooled Methylated Xanthine Compounds. *Phys. Chem. Chem. Phys.* **2017**, *19* (33), 22375–22384. https://doi.org/10.1039/C7CP03380J.

(11) Plekan, O.; Feyer, V.; Richter, R.; Moise, A.; Coreno, M.; Prince, K. C.; Zaytseva, I. L.; Moskovskaya, T. E.; Soshnikov, D. Yu.; Trofimov, A. B. X-Ray Spectroscopy of Heterocyclic Biochemicals: Xanthine, Hypoxanthine, and Caffeine. *J. Phys. Chem. A* **2012**, *116* (23), 5653–5664. https://doi.org/10.1021/jp300459p.

(12) Platonov, M. O.; Samijlenko, S. P.; Sudakov, O. O.; Kondratyuk, I. V.; Hovorun, D. M. To What Extent Can Methyl Derivatives Be Regarded as Stabilized Tautomers of Xanthine? *Spectrochim. Acta. A. Mol. Biomol. Spectrosc.* **2005**, *62* (1–3), 112–114. https://doi.org/10.1016/j.saa.2004.12.012.

(13) Rogstad, K. N.; Jang, Y. H.; Sowers, L. C.; Goddard, W. A. First Principles Calculations of the p K_a Values and Tautomers of Isoguanine and Xanthine. *Chem. Res. Toxicol.* **2003**, *16* (11), 1455–1462. https://doi.org/10.1021/tx034068e.
(14) Lichtenberg, D.; Bergmann, F.; Neiman, Z. Tautomeric Forms and Ionisation Processes in Xanthine and Its N-Methyl Derivatives. *J. Chem. Soc. C Org.* **1971**, 1676. https://doi.org/10.1039/j39710001676.
(15) Dybiec, K.; Molchanov, S.; Gryff-Keller, A. Structure of Neutral Molecules and Monoanions of Selected Oxopurines in Aqueous Solutions As Studied by NMR Spectroscopy and Theoretical Calculations. *J. Phys. Chem. A* **2011**, *115* (10), 2057–2064. https://doi.org/10.1021/jp110888m.
(16) Emel'yanenko, V. N.; Zaitsau, D. H.; Verevkin, S. P. Thermochemical Properties of Xanthine and Hypoxanthine Revisited. *J. Chem. Eng. Data* **2017**, *62* (9), 2606–2609. https://doi.org/10.1021/acs.jced.7b00085.
(17) Pung, A.; Leito, I. Predicting Relative Stability of Conformers in Solution with COSMO-RS. *J. Phys. Chem. A* **2017**, *121* (36), 6823–6829. https://doi.org/10.1021/acs.jpca.7b05197.
(18) Dührkop, K.; Fleischauer, M.; Ludwig, M.; Aksenov, A. A.; Melnik, A. V.; Meusel, M.; Dorrestein, P. C.; Rousu, J.; Böcker, S. SIRIUS 4: A Rapid Tool for Turning Tandem Mass Spectra into Metabolite Structure Information. *Nat. Methods* **2019**, *16* (4), 299–302. https://doi.org/10.1038/s41592-019-0344-8.
(19) Dührkop, K.; Shen, H.; Meusel, M.; Rousu, J.; Böcker, S. Searching Molecular Structure Databases with Tandem Mass Spectra Using CSI:FingerID. *Proc. Natl. Acad. Sci.* **2015**, *112* (41), 12580–12585. https://doi.org/10.1073/pnas.1509788112.
(20) Böcker, S.; Dührkop, K. Fragmentation Trees Reloaded. *J. Cheminformatics* **2016**, *8* (1), 5. https://doi.org/10.1186/s13321-016-0116-8.
(21) Liigand, J.; Wang, T.; Kellogg, J.; Smedsgaard, J.; Cech, N.; Kruve, A. Quantification for Non-Targeted LC/MS Screening without Standard Substances. *Sci. Rep.* **2020**, *10* (1), 5808. https://doi.org/10.1038/s41598-020-62573-z.
(22) Kruve, A.; Kaupmees, K.; Liigand, J.; Leito, I. Negative Electrospray Ionization via Deprotonation: Predicting the Ionization Efficiency. *Anal. Chem.* **2014**, *86* (10), 4822–4830. https://doi.org/10.1021/ac404066v.
(23) Liigand, J.; Kruve, A.; Leito, I.; Girod, M.; Antoine, R. Effect of Mobile Phase on Electrospray Ionization Efficiency. *J. Am. Soc. Mass Spectrom.* **2014**, *25* (11), 1853–1861. https://doi.org/10.1007/s13361-014-0969-x.
(24) Kruve, A. Influence of Mobile Phase, Source Parameters and Source Type on Electrospray Ionization Efficiency in Negative Ion Mode: Influence of Mobile Phase in ESI/MS. *J. Mass Spectrom.* **2016**, *51* (8), 596–601. https://doi.org/10.1002/jms.3790.
